# Selective Sorption of Rhenium and Molybdenum Oxoanions Using an Interpolymer System Based on Functionalized Cellulose and Lewatit Monoplus SP112H in Acidic Aqueous Media

**DOI:** 10.3390/polym18161943

**Published:** 2026-08-08

**Authors:** Dametken Fischer, Sultan Yulusov, Arman Baishibekov, Talkybek Jumadilov, Józef Haponiuk, Saniya Temirova, Bagdat Altaibayev, Tatiana Surkova, Axaule Mamaeva, Kenzhegali Smailov

**Affiliations:** 1 Institute of Metallurgy and Ore Beneficiation JSC, Satbayev University, Shevchenko Str., 29/133, Almaty 050010, Kazakhstan; s.yulussov@satbayev.university (S.Y.); abayshibekov@gmail.com (A.B.); s.temirova2401@gmail.com (S.T.); b.altaibayev@satbayev.university (B.A.); t.surkova@satbayev.university (T.S.); ak78@mail.ru (A.M.); k.smailov@satbayev.university (K.S.); 2 A.B. Bekturov Institute of Chemical Sciences JSC, Satbayev University, Sh. Ualikhanov Str., 106, Almaty 050010, Kazakhstan; jumadilovtalkybek@gmail.com; 3 Polymer Technology Department, Faculty of Chemistry, Gdańsk University, 11/12 Gabriela Narutowicza Street, 80-233 Gdańsk, Poland; jozef.haponiuk@pg.edu.pl

**Keywords:** rhenium, molybdenum, cellulose-PEI-GA, interpolymer system, anion exchange, sorption, hydrometallurgy, Schiff base crosslinking, Lewatit MonoPlus SP112H, sustainable recovery, circular economy

## Abstract

Isolating rhenium and molybdenum selectively from multicomponent acidic hydrometallurgical solutions remains problematic because of the abundance of competing cations and the low concentration of target oxoanions. This study investigates the sorption of Re(VII) and Mo(VI) oxoanions by an interpolymer system (IPS) comprising a cellulose–polyethyleneimine–glutaraldehyde (Cellulose-PEI-GA) weak-base anion exchanger and a sulfonated styrene–divinylbenzene cation-exchange resin (Lewatit MonoPlus SP112H), operating as spatially separated but solution-coupled phases. The industrial feed, a sulfuric acid leach liquor from electrostatic precipitator dust at the Zhezkazgan copper smelter, Kazakhstan (pH 1.25), was pretreated by liquid–liquid extraction with a trialkylamine-2-ethylhexanol–kerosene system followed by ammonia stripping to give an alkaline re-extract (pH 11.98) used for sorption. At the optimal 1:1 mass ratio of the two components (3:3), the IPS achieved a rhenium recovery of 77.7% (Kd = 3678 mL g^−1^, q = 4.51 mg g^−1^) against a markedly lower molybdenum recovery of 24.0% (Kd = 333 mL g^−1^), giving a Re/Mo separation factor β of 8.8–15.5 across the studied compositions; this uptake exceeded the value predicted from the individual sorbents by a factor of ~2.6, indicating a cooperative rather than purely additive effect. Sorption kinetics (pseudo-first-order, pseudo-second-order and Elovich models) and diffusion mechanisms (Weber–Morris and Boyd models) were consistent with mixed film- and intraparticle-diffusion control. Desorption with hydrochloric acid recovered 91.4% of the sorbed rhenium and 89.1% of the molybdenum, and FTIR, TGA, and BET analyses (surface area increasing from 1.93 to 8.62 m^2^ g^−1^ for the cellulose component and from 7.21 to 8.46 m^2^ g^−1^ for the resin) confirmed sorbate-induced textural and compositional changes consistent with the proposed complementary anion-/cation-exchange mechanism.

## 1. Introduction

The high hydration energies, similar structural features, and competition with background electrolytes make selective sorption of metal oxoanions from acidic aqueous media difficult [1,2,3]. In such systems, rhenium and molybdenum are usually in the form of perrhenate, ${ReO}_{4}^{-}$, and molybdate, ${MoO}_{4}^{2-}$, or their protonated forms, set by the acidity and the background electrolyte level [4,5]. At a low pH (<4), Mo(VI) predominantly forms highly charged polymolybdate species (e.g., Mo_7_$O_{24}^{6-}$); above pH~7 it converts to the simple molybdate ion Mo$O_{42}^{-}$ (see Supplementary Table S2 for the full pH-dependent speciation). For efficient separation of these species, sorbents are needed that contain functional groups active under acidic conditions and are capable of binding electrostatically, via proton-assisted interactions, and, in some cases, via specific donor–acceptor interactions [1,6].

Polymeric sorbents and ion-exchangers represent some of the most studied media for oxoanion separation and preconcentration [6,7,8]. The ${ReO}_{4}^{-}\;$ affinity is observed for both strong- and weak-base anion exchangers, but their selectivity is often reduced in multicomponent acidic solutions due to competition for sorption sites, diffusion limitations, and variations in metal speciation [4,5,6]. Anion-exchange resins have proven effective for the recovery of molybdenum oxoanions from sulfate leach liquors; for example, the weakly basic resin Lewatit MP62W5 achieved over 88% molybdenum sorption at pH 5.5, with 93–94% desorption by ammonium sulfate [9]. Other classes of sorbents have been studied, mostly in model systems, where relatively high uptake values are often reported [10,11,12,13], such as activated carbon, biochar-derived materials, mesoporous silicas, layered double hydroxides, and functionalized porous frameworks. However, in many cases only the sorption capacity has been considered, while less attention has been paid to the effects of polymer architecture, functional group arrangement and cooperative interactions between complementary sorption phases [1,14].

Cellulose is an attractive matrix for the design of hybrid sorbents, due to its availability, mechanical stability, chemical resistance and high density of hydroxyl groups readily amenable to chemical modification [15,16,17,18]. The functionalization of cellulose with amine-containing polymers allows for the synthesis of structurally stable sorbents with tunable surface chemistry and a controllable density of active sites [8,19,20,21,22]. Among these modifiers, polyethyleneimine (PEI) is especially attractive due to its macromolecular structure rich in primary, secondary and tertiary amino groups, which can be readily protonated in acidic media; upon protonation these groups become active sites that attract anionic metal species via Coulombic interactions and anion-exchange mechanisms [8,19,20,21,22,23,24,25].

The typical approach for stabilization on a cellulose surface involves crosslinking of PEI with glutaraldehyde (GA). Covalent imine (Schiff base) bonds are formed involving the aldehyde groups of GA and the primary and secondary -NH groups of PEI, preventing polymer leaching while anchoring the functional layer to the cellulose matrix [8,24,25,26]. The produced cellulose-PEI-GA composite can be considered as a stable weak-base anion-binding material with a high density of accessible protonatable amino groups [8,26]. The Schiff base crosslinking strategy has been widely employed in the design of PEI-functionalized cellulose composites such as aerogels and microgels, which provides both structural integrity and antimicrobial or adsorptive functionality [24,25,26]. Thus, the GA-to-PEI molar ratio defines the density of protonable sites and the crosslink degree and the accessibility of the PEI network, and glutaraldehyde crosslinking is a critical factor to tailor the anion-exchange capacity and the mechanical stability of the sorbent [24,26]. At the same time, there has been increased interest in interpolymer systems, in which two polymer phases with complementary ionic functions operate in a common medium [27,28,29,30,31,32], in addition to single component sorbents. In such systems, co-located cation- and anion-exchange resins within one solution undergo mutual activation through remote interaction: conformational and electrochemical changes in each polymer network change the ionization state of both components, leading to sorption performance exceeding that of the individual phases [27,28,29,30,31]. This cooperative effect has been demonstrated for the recovery of rare-earths, precious metals and transition-metal ions from complex multicomponent solutions, where interpolymer systems based on industrial ion exchangers always show a higher sorption capacity, selectivity and regenerability than a single sorbent system [27,28,29,30,31]. The interpolymer approach was also shown to be applicable to rhenium for PAA:P4VP systems, confirming the composition ratio of the polymer pair as a key parameter governing sorption efficiency [32]. For the present work, the amine-functionalized cellulose composite is mixed with Lewatit MonoPlus SP112H, a strong-acid cation exchanger built from a crosslinked styrene–divinylbenzene skeleton functionalized with sulfonic acid groups, -SO_3_H [33]. In this pair, the cellulose-PEI-GA composite is mainly an anion-binding phase through its protonatable amino groups, while the sulfonated resin provides a strongly acidic local environment, and could also be used to reduce the concentration of multivalent cations competing in solution [27]. This functional differentiation is anticipated to influence solution composition and protonation equilibria in different but complementary ways. The proposed system is characterized by the fact that the two sorbents are placed in a common volume of solution but were physically separated into individual permeable bags. Under these conditions the system may be viewed as an interpolymer pair, spatially separated, with indirectly coupled functionalities [29]. Such an arrangement excludes mechanical mixing of the phases, simplifies their subsequent regeneration, and at the same time allows the surrounding electrolyte to mediate redistribution of ionic species between the two polymer domains. Therefore, local protonation equilibria, ion competition, and diffusion regimes may be different from those observed for each sorbent used separately [1,5]. This is especially true for real industrial solutions. In contrast to simplified model media, hydrometallurgical sulfuric acid liquors resulting from the lead dust processing of the Zhezkazgan copper smelter contain not only the target rhenium and molybdenum species, but also considerable amounts of competing cations and background electrolytes [34]. The sorption behavior in these systems is affected by ionic strength, sulfate concentration, proton activity, and competitive interaction for active sites and hydration shells [2,5]. Therefore, the performance of a sorbent cannot be properly assessed on the basis of model solutions only.

This is particularly true of the sulfate leach liquors of electrostatic precipitator dust at the Zhezkazgan smelter. Their composition is characterized by a rhenium content of about 20–25 mg L^−1^ in the presence of Cu (about 29.7 g L^−1^), Zn (about 50.7 g L^−1^), Fe (about 2.3 g L^−1^), Mo (about 143 mg L^−1^), As, and Pb with a total sulfate content of about 118 g L^−1^. The strong competitive burden of transition-metal cations severely suppresses the direct sorption of rhenium under these conditions. Rhenium has also been recovered from such copper-production lead sludges by pyrometallurgical routes, for example oxidative roasting with calcium hypochlorite to form calcium perrhenate followed by water leaching, with rhenium recoveries reaching 93–94% [35]. Therefore, a liquid–liquid extraction step with a trialkylamine-2–ethylhexanol–kerosene system is used as a pre-treatment (purification) step: ${ReO}_{4}^{-}$ is extracted selectively into the organic phase while most of the competing cations and some arsenic and lead are precipitated at the phase boundary as insoluble sulfate-arsenate compounds [34,35,36]. In a subsequent step, the loaded organic phase is stripped with aqueous ammonia, leading to an ammoniacal re-extract with very low concentrations of interfering metals, which is then directed to sorption on ion-exchange materials. This two-step process of solvent extraction and sorption is a general approach in rhenium hydrometallurgy and serves as a basis for the evaluation of sorbent performance under conditions close to industrial [36,37].

Cellulose-based sorbents and traditional ion-exchange resins have been well studied as individual materials, but their behavior in spatially separated chemically complementary interpolymer configurations has not been sufficiently examined [27,28,29,30,31,32]. Specifically, the influence of such systems on protonation equilibria, diffusion-controlled sorption, and selectivity of Re(VII) and Mo(VI) oxoanions in strongly acidic multicomponent media is poorly understood [1,6,38]. Therefore, the individual sorbents need to be compared with their interpolymer system to determine whether cooperative interactions between the polymer phases cause measurable changes in sorption kinetics, equilibrium parameters, and regeneration behavior. In the current work, the sorption of rhenium and molybdenum oxoanions by an interpolymer system based on cellulose-PEI-GA and a cation exchanger based on sulfonated styrene–divinylbenzene (Lewatit MonoPlus SP112H) was studied.

Kinetic modelling is widely used to identify the kinetically limiting stage and the nature of sorbate–sorbent interactions. The pseudo-first-order (PFO) and pseudo-second-order (PSO) models are routinely applied to describe metal- and oxoanion-uptake kinetics on functionalized biopolymer and cellulose-based sorbents [39,40,41], while the Elovich equation accounts for chemisorption on energetically heterogeneous surfaces [41,42]. To establish whether film or intraparticle diffusion controls the rate, the Weber–Morris and Boyd models are commonly employed; when the Weber–Morris plot fails to pass through the origin and the Boyd plot is non-linear, surface sorption and intraparticle diffusion are taken to act at the same time [43,44,45]. The sorption of rhenium from the re-extract of fixed composition was interpreted through these kinetic and diffusion models rather than through equilibrium isotherms.

The sorption kinetics and diffusion behavior are evaluated together with the selectivity and regeneration characteristics of the system and the participation of functional groups, assessed by FTIR and thermogravimetric analysis [46,47,48,49,50,51,52,53,54]. For acidic multicomponent systems, the results are expected to shed light on the role of complementary polymer components in oxoanion binding and to provide a basis for the development of regenerable sorption systems for selective isolation of rhenium and molybdenum from multicomponent aqueous media.

Against this background, this study concentrates on selectively recovering rhenium, the target critical metal, from the ammoniacal re-extract obtained after solvent-extraction pre-treatment of the Zhezkazgan copper-smelter liquors. Because rhenium and molybdenum are geochemical companions, rhenium is isomorphically incorporated in molybdenite and is therefore inevitably accompanied by molybdenum in copper- and molybdenum-processing solutions; the two oxoanions cannot be considered in isolation. Molybdenum is consequently treated throughout this work as the principal co-occurring oxoanion against which the selectivity of the interpolymer system is assessed, rather than as a second target of equal priority. Accordingly, the characterization of the sorbent and the optimization of the sorption conditions are centered on rhenium uptake, while molybdenum is monitored in parallel to quantify the Re/Mo separation achieved by the system.

The novelty of this work lies in the use of a spatially separated Cellulose-PEI-GA: Lewatit MonoPlus SP112H interpolymer system for the selective recovery of rhenium from a genuine industrial copper-smelter solution, rather than from model media. For the first time, the complementary roles of the two phases are established directly: FTIR reveals the characteristic perrhenate Re=O band at 894.9 cm^−1^ on the amine-functionalized cellulose but not on the strong-acid resin, demonstrating that the anionic rhenium species is bound by the cellulose component, while SEM-EDS elemental analysis confirms the retention of competing cations on the resin. The study further demonstrates a two-level selectivity concept, in which solvent-extraction pre-treatment and interpolymer sorption act in series to achieve a high Re/Mo separation factor under conditions close to industrial practice.

## 2. Materials and Methods

### 2.1. Reagents, Materials and Preparation

#### Materials

The interpolymer sorption system (IPS) comprised two components: (1) a modified cellulose sorbent functionalized with polyethyleneimine (PEI) (Sigma-Aldrich Chemie GmbH, Taufkirchen, Germany) and crosslinked with glutaraldehyde (GA) (Sigma-Aldrich (Switzerland) Holding Ag, Buchs, Switzerland) (hereafter Cellulose–PEI–GA); and (2) the commercial strong-acid cation-exchange resin Lewatit MonoPlus SP112H (Lanxess/Tianjin Shengtai Chemical Co., Ltd., Nankai District, Tianjin, China) based on a styrene–divinylbenzene copolymer with covalently bound sulfonate groups (-SO_3_H) and a nominal exchange capacity of ~1.8 meq/mL. Microcrystalline cellulose (average particle size 50–100 μm, Sigma-Aldrich Trading Co., Ltd., Shanghai, China) was used as the polymer matrix. Polyethyleneimine (PEI, 50 wt.% aqueous solution, branched, $\bar{M}$w~750.000, Sigma-Aldrich) and a 25 wt.% aqueous solution of glutaraldehyde (GA, Sigma-Aldrich) were employed directly without additional treatment. For the liquid–liquid extraction step, an organic phase based on trialkylamine (TAA, 10 vol.%, 0.2 mol L^−1^) in aviation kerosene with 2-ethylhexanol as a phase modifier was used. Rhenium was recovered from the loaded organic phase by stripping with a 1:1 aqueous ammonia solution (NH_3_ aq.). Analytical-grade reagents: sulfuric acid (H_2_SO_4_, 98%, reagent grade), sodium hydroxide (NaOH), ammonium hydroxide (NH_4_OH), acetic acid (CH_3_COOH) were sourced from Sigma-Aldrich or local chemical suppliers. Deionized water was applied in every experimental procedure.

### 2.2. Industrial Feed Solution

The primary object of investigation is an industrial sulfuric acid leach solution (hereafter ‘industrial solution’) produced at Polymettech LLP (Shymkent, Kazakhstan) by hydrometallurgical processing of lead dust generated at the Zhezkazgan copper smelter. The solution exhibits a characteristic blue color arising from high Cu^2+^ content and an acidic reaction medium (pH 1.25). The ammoniacal re-extract obtained in the subsequent stage has pH 11.98. Although the industrial solution treated in this study is strongly acidic (pH 1.25), consistent with the “acidic aqueous media” referenced in the title, the sorption step itself is carried out from the alkaline ammoniacal re-extract (pH 11.98) obtained after the extraction–stripping pretreatment; the acidic character therefore refers to the origin of the treated industrial medium rather than to the sorption stage.

#### 2.2.1. Plant-Scale Process for Solution Production

**Figure A1:**
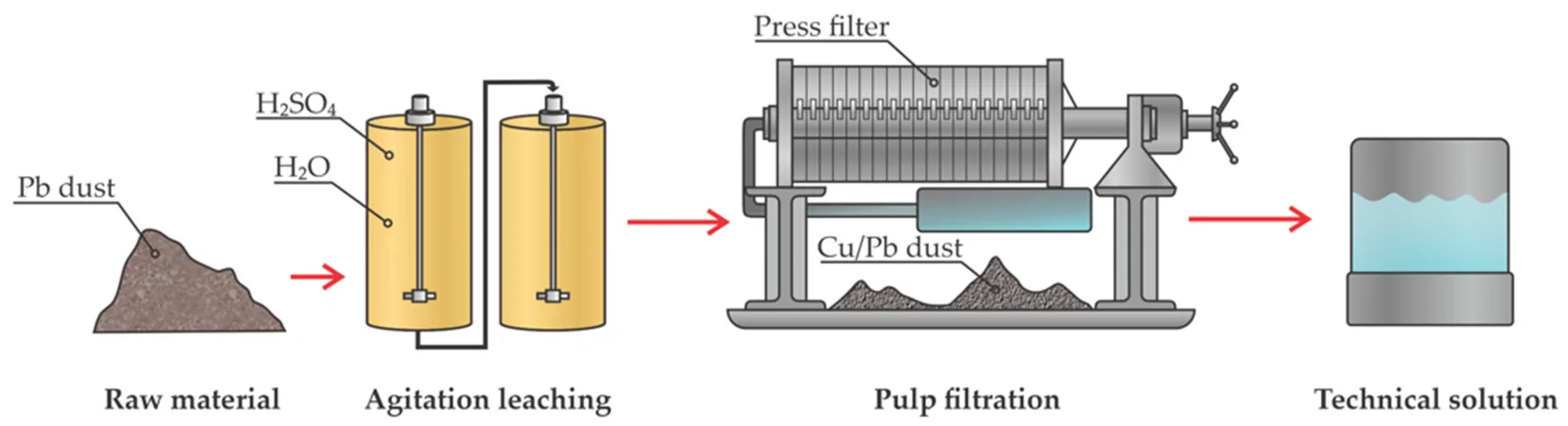
Technological scheme for obtaining a Cu–Re–Mo-bearing technical solution from lead dust through sulfuric acid leaching and subsequent pulp filtration.

Lead dust is generated during pyrometallurgical processing of copper sulfide concentrates and collected by gas-cleaning systems of smelting and converting units as fine-grained technogenic feed. The industrial solution is obtained by sulfuric acid leaching of lead dust in agitation tanks; Cu, Zn, Re, Mo, Bi, Cd and other metals are transferred into solution as sulfates, while the bulk of lead is concentrated in the solid phase. The leach slurry is separated from filter-press equipment, yielding a concentrate enriched in lead (~50% Pb) and a copper sulfate liquor. The latter is subjected to cementation precipitation of copper with iron powder, producing a copper concentrate (~75% Cu) and a tailing solution directed to neutralization. A simplified flowsheet of industrial solution production from lead dust is shown in Figure A1. (Appendix A). The process includes sulfuric acid agitation leaching of lead dust, separation of the resulting pulp by filter pressing, and recovery of a metal-bearing technical solution for subsequent hydrometallurgical processing.

#### 2.2.2. Component Analysis of the Industrial Solution

The chemical compositions of the industrial sulfuric acid solution and lead dust were determined by inductively coupled plasma optical emission spectrometry (ICP–OES) using an Optima 8300 DV spectrometer (PerkinElmer, Shelton, CT, USA). The concentrations of major and minor components in the industrial solution are presented in Table 1 and Table 2.

##### Compositional Analysis

The industrial solution is a complex multi-component sulfuric acid system with high concentrations of Zn (50.7 g L^−1^), Cu (29.7 g L^−1^), and ${SO}_{4}^{2-}$ (128.5 g L^−1^). The target species, rhenium (22.73 mg L^−1^) and molybdenum (143.26 mg L^−1^), are present against a large excess of ballast cations. The high content of P (3.65 g L^−1^) and ${SO}_{4}^{2-}$ introduces competition in anion sorption, while the acidic medium (pH 1.25) and Fe^2+^/Fe^3+^ content (2.31 g L^−1^) are typical of acid copper-plant effluents.

#### 2.2.3. Preparation of the Feed Solution for Sorption Experiments

The industrial sulfuric acid solution served as the initial feed for solvent extraction. Following extraction, the loaded organic phase was subjected to stripping to recover the target metals into an aqueous phase. The re-extract with 5.49 mg L^−1^ Re and 71.16 mg L^−1^ Mo was used as the feed solution for the sorption experiments on the interpolymer system. Therefore, the proposed process integrates solvent extraction with sorption to achieve purification and selective recovery of rhenium and molybdenum.

#### 2.2.4. Lead Dust Raw Material Composition

The composition of the lead dust was determined by two complementary analytical techniques. The elemental composition was rapidly semi-quantitatively characterized by X-Ray fluorescence spectrometry (XRF) (Table 3). For quantitative elemental analysis, the lead dust samples were first acid digested to transfer the solid material into solution. The resulting solutions were analyzed by inductively coupled plasma optical emission spectrometry (ICP-OES) using an Optima 8300 DV spectrometer (PerkinElmer, Shelton, CT, USA).

The quantitative elemental composition obtained by ICP-OES is presented in Table 3. The elemental contents obtained by XRF (Table 3) and ICP-OES (Table 4) differ for several elements, especially Pb (52.99 vs. 27.98 wt.%), Cu (4.18 vs. 9.84 wt.%), and Fe (0.64 vs. 5.23 wt.%). They are the consequence of different analytical principles underlying the two methods. XRF is a rapid, semi-quantitative technique whose results may be affected by matrix effects, particle-size distribution, sample heterogeneity, and limited sensitivity for light elements. In contrast, ICP–OES determines the bulk elemental composition after complete dissolution of the sample, providing more accurate quantitative concentrations of major and minor elements. Therefore, XRF was used to obtain a rapid overview of the elemental composition of the lead dust, whereas the ICP-OES results were used as the quantitative bulk composition for subsequent calculations and discussion throughout this work. The close agreement observed for zinc (3.92 and 3.83 wt.%) confirms the consistency of the two analytical datasets.

Rhenium (0.0211%) and molybdenum (0.138%) present in the lead dust are transferred into solution during sulfuric acid leaching. The high content of S (14.35%), As (2.85%), Cl^−^ (0.11%) and P (0.21%) provides a substantial background of competing anions for the subsequent sorption experiments.

### 2.3. Synthesis of the CellulosePEI–GA Sorbent

**Figure 1:**
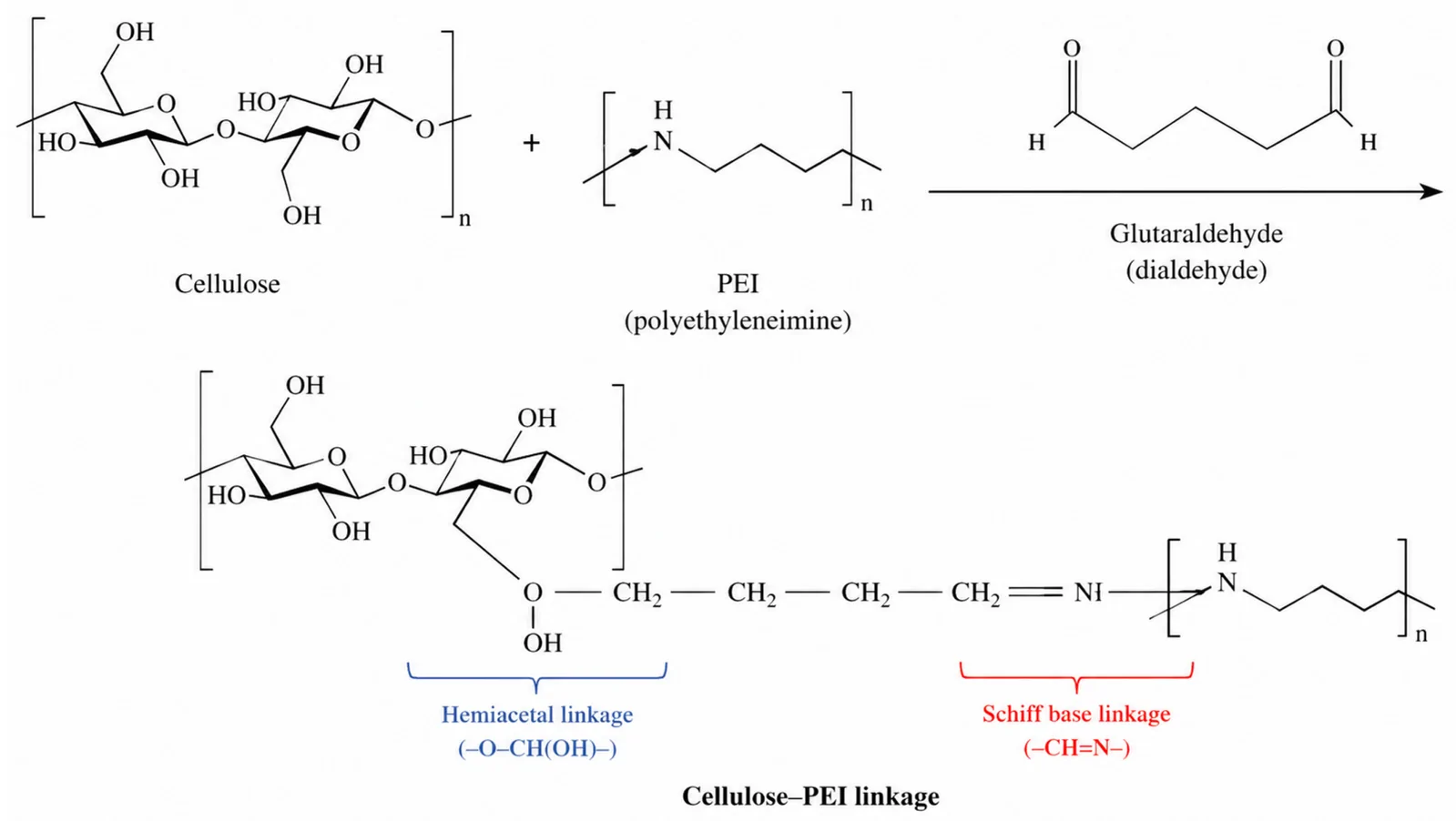
Schematic representation of cellulose-PEI-GA composite formation via glutaraldehyde crosslinking.

The Cellulose-PEI-GA sorbent was synthesized by immobilizing polyethyleneimine on the cellulose surface followed by covalent crosslinking with glutaraldehyde through Schiff base (imine) bond formation. A 5.0 g portion of dry cellulose powder was homogeneously suspended in 50 mL of deionized water with continuous mechanical agitation. A 50 wt.% aqueous PEI solution (10 mL) diluted in 10 mL of water was introduced dropwise, and the resulting suspension was continuously stirred for 30 min at ambient temperature; when required, the pH was brought to 8 by dropwise addition of dilute NaOH. Subsequently, 3 mL of a 25 wt.% aqueous GA solution diluted in 10 mL of water was added dropwise under continuous agitation. The crosslinking reaction was maintained for 1 h at 25 °C. The solid was recovered by filtration and washed with successive portions of deionized water until the filtrate approached neutral pH and dried in a laboratory oven at 60 °C to constant weight. The dry cake was milled and size-fractionated to obtain a particle fraction of 0.1–0.5 mm. For activation, a 5 g portion of the sorbent was stirred with 0.5 M acetic acid for 1 h, rinsed with deionized water until the eluate reached pH 5–6, and dried. The proposed mechanism of Cellulose-PEI-GA sorbent formation is presented in Figure 1. During the synthesis, glutaraldehyde as a bifunctional crosslinker links the cellulose hydroxyl groups with the amino groups of polyethyleneimine. The resulting structure contains hemiacetal linkages formed between cellulose and glutaraldehyde, as well as Schiff base (imine) bonds formed through the reaction of aldehyde groups with PEI amino groups, leading to the formation of a stable cellulose–PEI network.

### 2.4. Liquid–Liquid Extraction of Rhenium from the Industrial Solution

#### 2.4.1. Feed Solution for Extraction

The sulfuric acid leach solution of lead dust (Table 1) was directed to the extraction stage. The solution was blue with slight turbidity and contained 22.73 mg L^−1^ Re and 143.26 mg L^−1^ Mo against a large excess of base-metal cations (Cu, Zn, Fe) and sulfate (Table 1).

#### 2.4.2. Extractant Preparation

The organic phase was prepared by mixing aviation kerosene, trialkylamine (TAA), and 2-ethylhexanol as a phase modifier. The TAA concentration was 10 vol.% (0.2 mol L^−1^).

#### 2.4.3. Extraction Conditions

Extraction was carried out in a separatory funnel at an organic-to-aqueous phase ratio of O:A = 1:10 (100 mL organic:1000 mL feed solution), with a 5 min contact time at 25 °C. Phase separation occurred within approximately 10–15 min, but an interfacial suspension (“crud”) remained entangled in the organic phase at the phase boundary; the system was therefore left to settle for 24 h to allow clean phase separation. After settling, the loaded organic phase (top), the crud (a white-bluish interfacial layer), and the raffinate were distinguishable, and the loaded organic phase and the raffinate were filtered through “blue ribbon” filter paper; the loaded organic phase was yellow with a bluish tint, while the raffinate was blue with slight turbidity. The recovered volumes were 73 mL of loaded organic phase and 975 mL of raffinate. An 8 mL aliquot of the loaded organic phase was taken for analytical control, leaving 65 mL that was carried forward to the stripping (re-extraction) stage.

#### 2.4.4. Crud Composition

The interfacial suspension (“crud”) entangled in the loaded organic phase was filtered off, dried at 105 °C, and analyzed by X-Ray fluorescence spectrometry (XRF). The results are given in Table 5.

The crud is dominated by Cu, Zn, and As, together with S, indicating co-precipitation of base-metal sulfates/arsenates at the phase boundary; rhenium is present only in a minor amount (0.119 wt.%), consistent with the low fraction of rhenium retained in the crud relative to the loaded organic phase.

#### 2.4.5. Re-Extraction of Rhenium

Stripping of rhenium from the loaded extract was performed with a 1:1 aqueous ammonia solution at an organic-to-aqueous ratio of 1:15 (65 mL organic: 975 mL ammonia), contact time 5 min, temperature 25 °C. Phase separation was complete within 10 min; after a 24 h settling period, the stripped organic and re-extract were filtered off. The striped organic was light yellow with a reddish tint; the re-extract was pale yellow. Volumes: stripped organic—65 mL, re-extract—975 mL.

#### 2.4.6. Analytical Control

Rhenium in all fractions was determined by: (1) chemical method (ASIPI); (2) spectrophotometry (PE-5400UV, λ = 450 nm); and (3) visual colorimetry (Egerts tubes). Preliminary oxidation of samples was applied to eliminate interference from Cu, Zn, Fe, and As. The corresponding data are given in Table 6.

Based on these concentrations, the extraction efficiency (feed to raffinate) was 77.6%, the stripping efficiency (loaded organic to re-extract) was approximately 52.5%, and the overall rhenium recovery from the industrial feed to the re-extract was approximately 23.6%. The enrichment factor (C _re-extract_/C_feed_) was approximately 0.24, indicating that the extraction–stripping sequence does not concentrate rhenium but instead serves to remove the bulk of competing cations (Cu, Zn, Fe) prior to sorption.

The ammoniacal re-extract with 5.494 mg L^−1^ Re and much lower amounts of interfering metals was employed as the feed solution for the subsequent sorption studies on the Cellulose-PEI-GA: Lewatit MonoPlus SP112H IPS. The residual impurities after extraction and re-extraction were mainly molybdenum (71.23 mg L^−1^), iron (1.284 g L^−1^), aluminum (0.167 g L^−1^), silicon (0.442 g L^−1^), sodium (0.116 mg L^−1^), and sulfate ions (52.685 g L^−1^), indicating efficient separation of the majority of accompanying metal impurities in the extraction–re-extraction process. Model solutions were prepared by dissolving ammonium perrhenate (NH_4_ReO_4_) and ammonium heptamolybdate ((NH_4_)_6_Mo_7_O_24_·4H_2_O) in 0.5–1.5 M H_2_SO_4_ to imitate the controlled conditions.

### 2.5. Assembly of the Interpolymer System

In interpolymer system experiments, equal masses of Cellulose-PEI-GA and Lewatit MonoPlus SP112H (0.5 g each, total sorbent loading 1.0 g) were packed into individual polypropylene mesh bags (~50 μm pore size) and submerged concurrently into the same 250 mL vessel, corresponding to a total dose of 4 g L^−1^. The two sorbents had no direct contact; both components shared the same solution, enabling assessment of their mutual influence on sorption.

### 2.6. Sorption Experiments

Sorption tests were conducted in 250 mL glass conical flasks with orbital shaking at 150 rpm. All sorption and desorption experiments were performed in triplicate under the same conditions. The reported values are the average of three independent measurements (*n* = 3) with a standard deviation below 2%. The results are presented in Table 7.

### 2.7. Calculated Parameters

The sorption capacity at a given time t ($q_{t}$) and at equilibrium (q_e_) was determined using:

(1) $$q_{t}=\frac{{(C}_{0}-C_{t})V}{m}$$

(2) $$q_{e}=\frac{\left(C_{0}-C_{e}\right)V}{m}$$

where C_0_, C_t_ and C_e_ stand for the rhenium levels (mg L^−1^) in the original solution, at time t and at equilibrium; V is the solution volume (L) and m the sorbent weight (g).

The Distribution Coefficient K_d_ Was Calculated as

(3) $$K_{d}=\frac{q_{e}}{C_{e}}$$

Selectivity for rhenium relative to molybdenum was expressed by the selectivity coefficient:

(4) $$K_{sel}=\frac{(q_{e,Re}/q_{e,Mo})}{(C_{e,Re}/C_{e,Mo})}$$

The desorption efficiency of rhenium and molybdenum was evaluated according to the following equation:

(5) $$H_{\mathrm{des}}\;(\%)=\;\frac{Cdes}{C0-Ce\times 100}$$

where C_0_ is the initial concentration of the metal in the aqueous re-extract before sorption (mg L^−1^); $C_{e}$ is the residual concentration of the metal after sorption (mg L^−1^); and $C_{des}$ is the concentration of the metal in the desorption solution (mg L^−1^).

Each experiment was carried out in three independent replicates, and the relative standard deviation of measured values did not exceed 5%.

### 2.8. Characterization and Analytical Methods

After sorption, all solid samples were separated by filtration, rinsed thoroughly with deionized water under identical conditions to the corresponding pristine (pre-sorption) samples, and dried using the same protocol, ensuring that the two sample sets differed only in their sorption history and not in washing or drying treatment.

The concentrations of Re, Mo, Cu, Zn, Fe and other elements in the industrial sulfuric acid solution were determined by inductively coupled plasma optical emission spectrometry (ICP-OES) using an Optima 8300 DV spectrometer (PerkinElmer, Shelton, CT, USA). Prior to ICP-OES analysis, lead dust samples were subjected to complete acid digestion to transfer the solid material into solution. The resulting solutions were analyzed using the same ICP-OES instrument. Calibration was performed using multi-element standard solutions.

The elemental composition of the lead dust (Table 3) and the solid precipitates formed during extraction were characterized by wavelength-dispersive X-Ray fluorescence (WDXRF) using an Axios 1 kW spectrometer (Malvern Panalytical, Almelo, The Netherlands).

Infrared spectra were collected on a Bruker ALFA II FT-IR spectrometer (Bruker, Shanghai, China) fitted with an ATR accessory over the spectral window of 4000–400 cm^−1^ at a spectral resolution of 4 cm^−1^ (64 scans accumulated per spectrum).

Thermal behavior of the samples was investigated by simultaneous thermogravimetric and differential thermal analysis (TG–DTA), with derivative thermogravimetry (DTG), using a NETZSCH STA 409 PC/PG simultaneous thermal analyzer (NETZSCH-Gerätebau GmbH, Selb, Germany). Approximately 10–20 mg of sample was heated from 25 to 600 °C at a heating rate of 10 °C min^−1^ under a nitrogen atmosphere (50 mL min^−1^). The TG, DTG, and DTA curves were recorded simultaneously to evaluate mass-loss behavior, thermal stability, and thermal effects associated with the decomposition of the samples.

Morphological analysis of the sorbents was carried out by scanning electron microscopy combined with energy-dispersive X-Ray microanalysis on a Quattro ESEM (Thermo Fisher Scientific Inc., Waltham, Massachusetts, USA) electron microscope.

Textural parameters: surface area, pore-size distribution and pore volume were assessed by low-temperature nitrogen adsorption–desorption on a Kubo ×1000 instrument (Xi’an Yima Opto-electrical Technology Co., Ltd., Xi’an, China), (77 K; outgassing at 105 °C for 6 h). The isotherms were processed with the BET, BJH, t-method, αs-method, DR, MP and HK models.

Solution pH during the sorption and desorption runs was tracked with a pH200EM REX pH meter (Inesa Instrument Rex, Shanghai, China); the pH of the ammoniacal re-extract remained essentially constant at ≈11.98 throughout the 168 h sorption period, with no significant drift between the initial and equilibrium values.

### 2.9. Kinetic and Diffusion Analysis

Sorption kinetic behavior was evaluated by fitting the measured uptake data to three rate models—pseudo-first-order (PFO), pseudo-second-order (PSO), and Elovich [46,47].

The PFO model is given by:

(6) $$q_{t}=q_{e}\left(1-e^{{-K}_{1}t}\right)$$

The PSO model is

(7) $$q_{t}=\frac{k_{2}q_{e}^{2}t}{1+k_{2}q_{e}t}$$

The Elovich model is

(8) $$q_{t}=\frac{1}{\beta }ln\left(1+\alpha \beta t\right)$$

where q_t_ denotes the loading per gram of sorbent at time t, q_e_ is the equilibrium capacity, and k_1_, k_2_, α, and β are kinetic parameters.

Intraparticle diffusion was analyzed using the Weber–Morris model [41]:

(9) qt=kid×t12+C

where q_t_ is the sorption capacity at time t (mg·g^−1^), k_id_ is the intraparticle diffusion rate parameter (mg·g^−1^·h ^(−1/2)^), t is the contact time (min), and C represents the intercept reflecting the boundary-layer effect (mg·g^−1^).

Diffusion mechanisms were evaluated using the Boyd model [42]:

(10) F=1−6π2×Σn=1→∞1n2×exp−n2Bt

where F = qtqe denotes the degree of approach to equilibrium, Bt is a mathematical function of F, and n is an integer (the summation index).

For the practical calculation of Bt from the experimental F values, the Reichenberg [49] approximations were used:

For F < 0.85: 2

(11) $$Bt=(\sqrt{\pi }-\sqrt{(\pi }-\frac{\pi^{2}\times F}{3}))$$

For F > 0.85:

(12) $$1Bt=-0.4977-ln\left(1-F\right)$$

The effective diffusion coefficient can be estimated from:

(13) $$B=\pi^{2}\times \frac{D_{i}}{r^{2}}$$

Here B is the constant obtained from the slope of the Bt–t plot (h^−1^), D_i_ is the effective diffusion coefficient (m^2^·h^−1^), and r is the sorbent particle radius (m).

## 3. Results and Discussion

The sorbent characterization (BET, SEM-EDS and FTIR) and the extraction–sorption study below are presented primarily with respect to rhenium, since rhenium is the target metal for which the interpolymer system was designed. Molybdenum, as the main accompanying oxoanion, is followed in the sorption experiments to evaluate the selectivity of the system rather than as an independent object of characterization.

### 3.1. BET Analysis of Pore Structure

#### 3.1.1. Textural Changes in Cellulose upon Modification and Sorption

**Figure 2:**
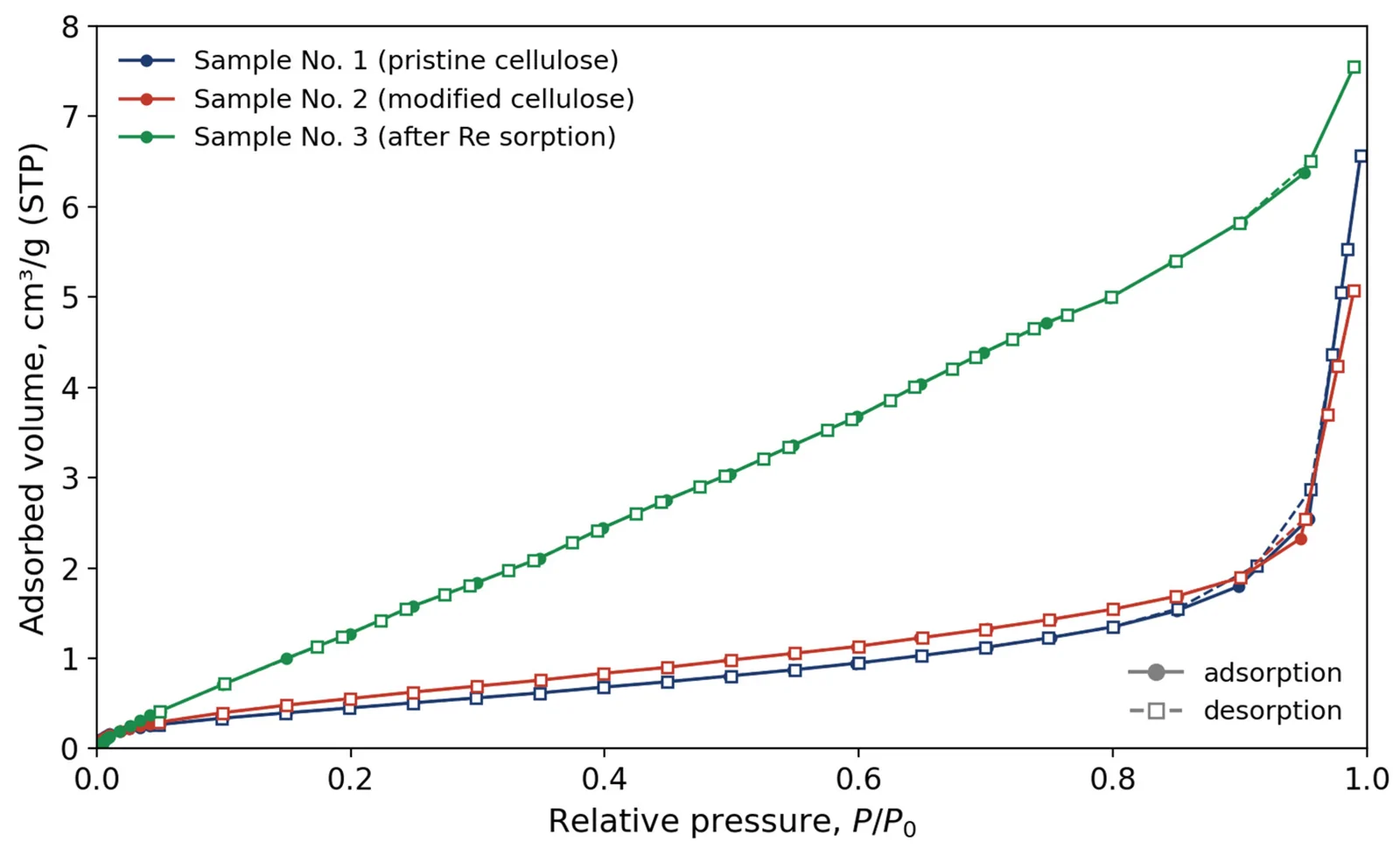
Cryogenic nitrogen adsorption–desorption isotherms (77 K) for Samples No. 1 (pristine cellulose), No. 2 (modified cellulose) and No. 3 (modified cellulose) (filled symbols—adsorption, open symbols—desorption).

**Figure 3:**
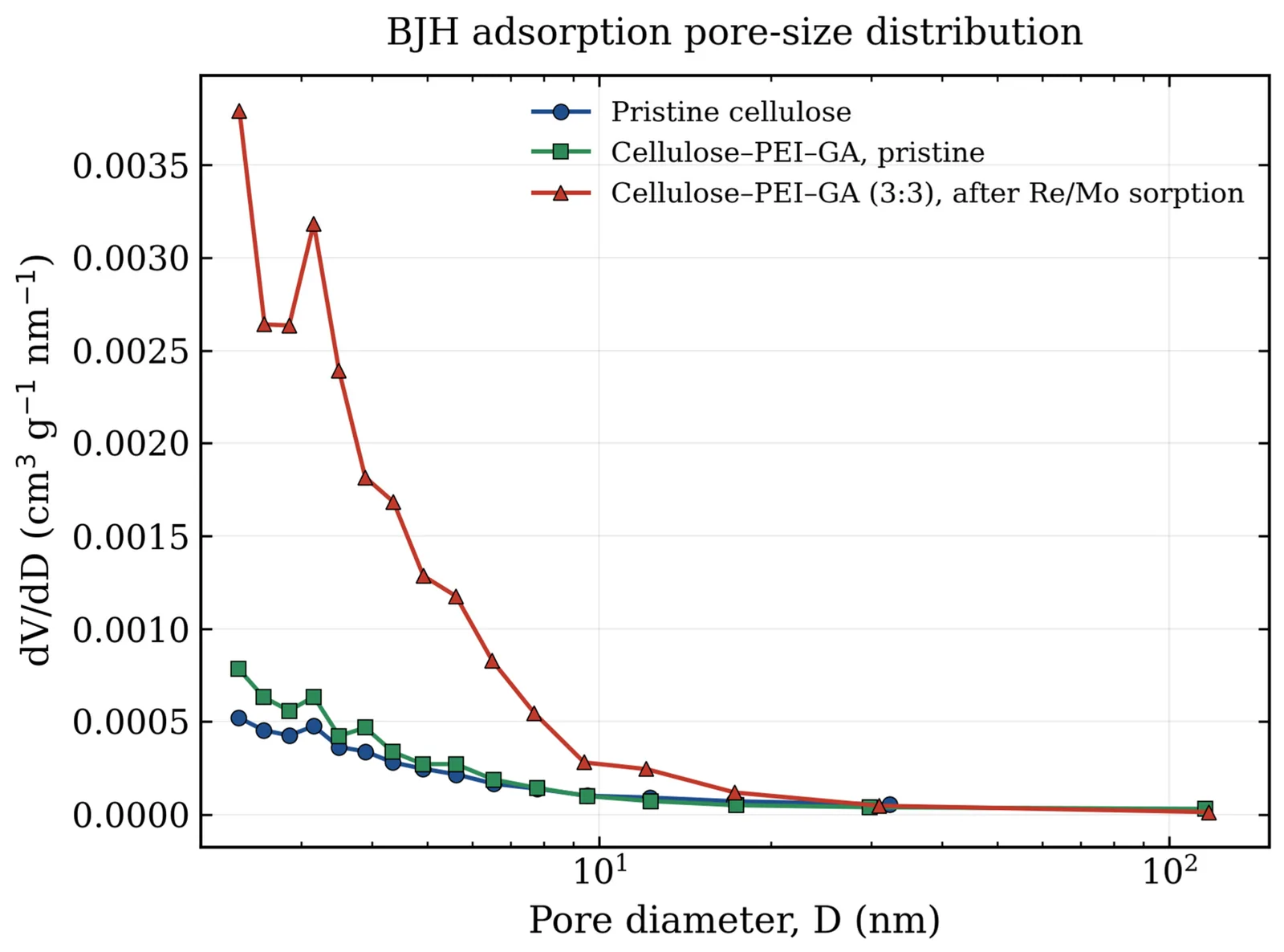
Pore size distribution (BJH, adsorption branch, dV/dlog(D)) for Samples No. 1, No. 2 and No. 3.

The porous structure of pristine cellulose (No. 1), modified Cellulose-PEI-GA (No. 2), and the modified sorbent after rhenium sorption from the re-extract (No. 3) was examined by low-temperature nitrogen adsorption–desorption (Kubo X1000, 77 K); the full set of textural parameters is given in Table S3 (Supplementary Materials). The BET surface area increased progressively across the series, from 1.93 m^2^/g (pristine cellulose) to 2.42 m^2^/g after PEI-GA functionalization (+25%, attributed to the anchored branched PEI network) and to 8.62 m^2^/g after rhenium sorption (a 3.6-fold increase over the modified sorbent), with a corresponding decrease in average BET pore radius (10.56 → 6.53 → 2.72 nm). The nitrogen adsorption–desorption isotherms (Figure 2) change from type II (samples No. 1–2, essentially non-porous/macroporous) to type IV with a pronounced capillary-condensation hysteresis loop (sample No. 3), and the BJH pore size distribution (Figure 3) shows the cumulative mesopore volume increasing ≈1.8-fold upon sorption, together indicating the development of a well-defined mesoporous structure driven by the sorption process itself rather than by the PEI-GA functionalization step alone. A contribution of physically deposited inorganic salts to the observed surface-area and mass gains, rather than solely a genuine increase in mesoporosity, cannot be fully excluded from BET and TGA data alone; however, all samples were washed and dried under identical conditions before and after sorption (Section 2.8), and the disproportionate gain concentrated specifically in the fine-mesopore range (2–10 nm), together with the isotherm-type transition and hysteresis development, is more consistent with genuine textural development during sorption than with simple salt crystallization on the external surface, which would be expected to reduce rather than increase the measured surface area.

#### 3.1.2. Textural Changes in Lewatit MonoPlus SP112H upon Sorption

**Figure 4:**
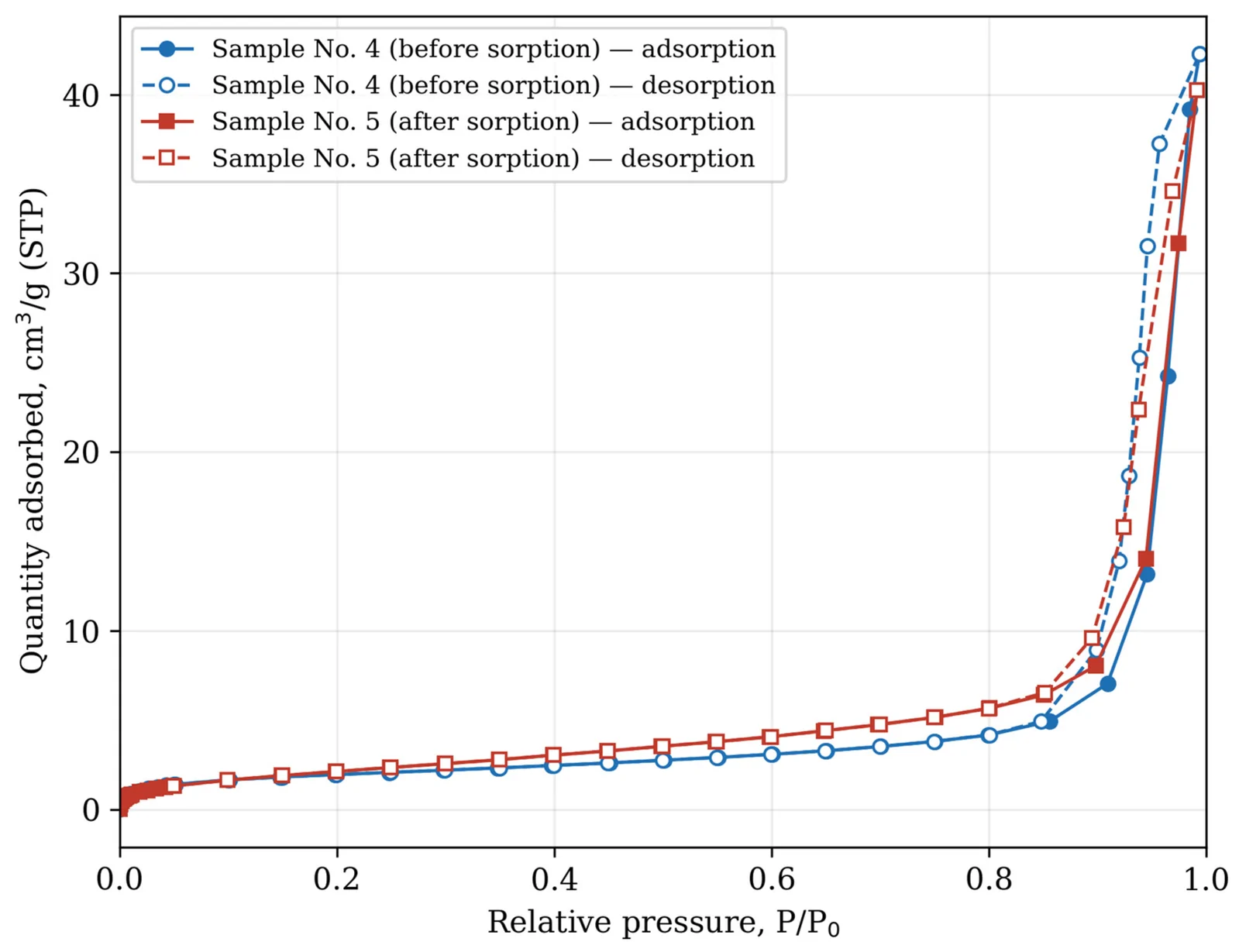
Cryogenic nitrogen adsorption–desorption isotherms (77 K) for Samples No. 4 (Lewatit MonoPlus SP112H, before sorption) and No. 5 (Lewatit MonoPlus SP112H, after sorption of Re(VII)/Mo(VI)) (filled symbols—adsorption, open symbols—desorption).

The porous structure of the strong-acid cation-exchange resin Lewatit MonoPlus SP112H (LANXESS Deutschland GmbH, Cologne, Germany; supplied by Tianjin Shengtai Chemical Co., Ltd., Tianjin, China), the second component of the interpolymer sorption system, was examined by low-temperature nitrogen adsorption–desorption (Kubo X1000, 77 K) before (Sample No. 4) and after (Sample No. 5) contact with the working solution; the full set of textural parameters is given in Table S4 (Supplementary Materials). The resin displays a coarse meso-/macroporous texture (BET surface area 7.21 m^2^/g before sorption, total pore volume ≈ 0.066 cm^3^/g, average pore radius ≈18–20 nm), typical of a macroreticular sulfonated styrene–divinylbenzene bead structure; t-plot and αs analyses confirm the complete absence of true micropores in both samples. Sorption of Re(VII) and Mo(VI) increases the BET surface area moderately, to 8.46 m^2^/g (+17%), while the total pore volume remains essentially unchanged (−5%). The nitrogen isotherms of both samples (Figure 4) correspond to type II with a pronounced H3-type hysteresis loop; sorption reduces the BET C constant substantially (78.3 → 28.1), indicating weaker adsorbate–surface interaction after surface loading with Re/Mo species.

**Figure 5:**
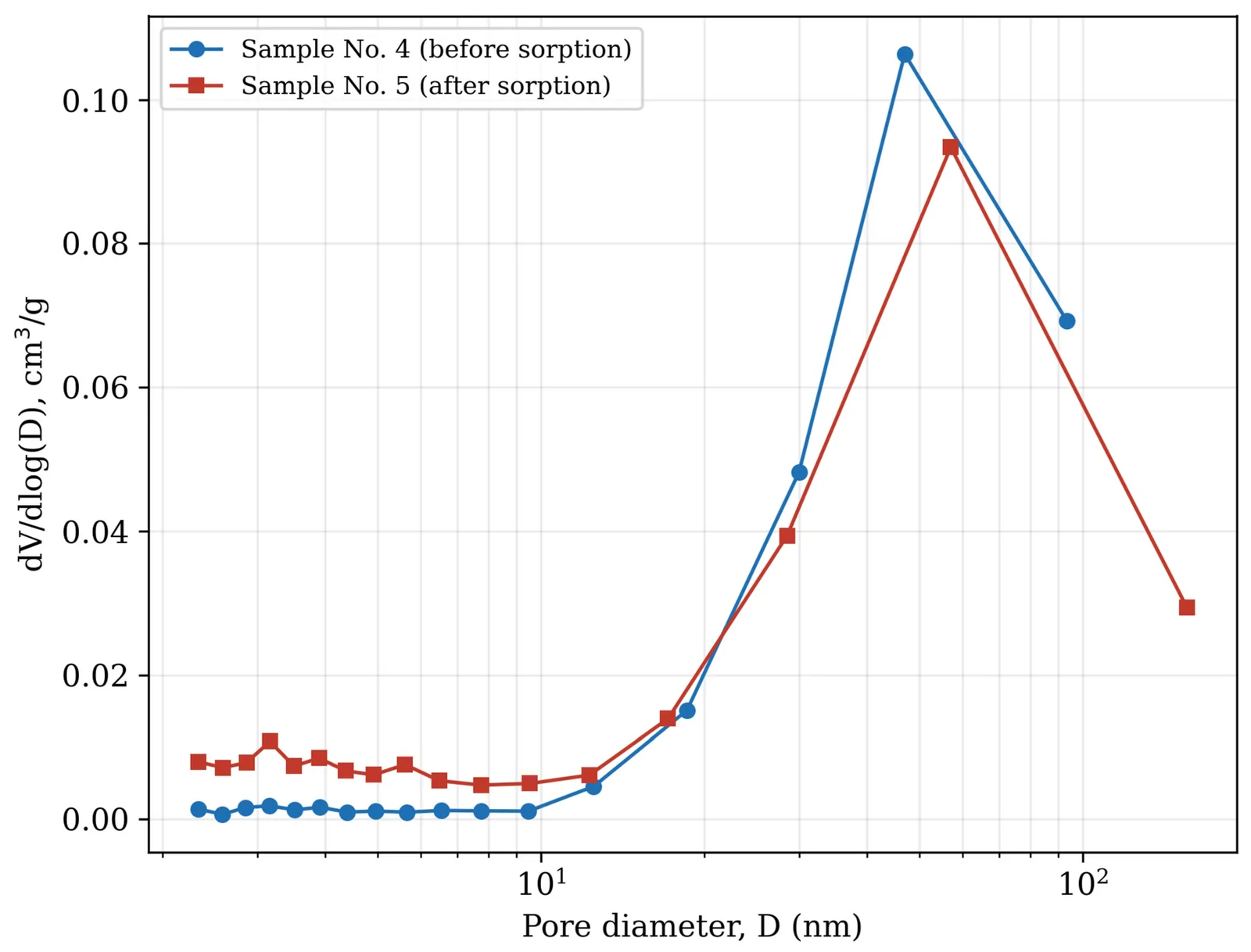
Pore size distribution (BJH, adsorption branch, dV/dlog(D)) for Samples No. 4 (Lewatit MonoPlus SP112H, before sorption) and No. 5 (Lewatit MonoPlus SP112H, after sorption).

The BJH pore-size distribution (Figure 5) shows that sorption causes a size-selective redistribution of porosity: the average pore radius contracts by ≈19–37%, and the fine-mesopore range (2–10 nm) gains intensity 5–6-fold, while the broad coarse-mesopore/macropore population (30–90 nm) is largely retained. The mesopore surface area (αs-method) shows the largest relative gain of all textural parameters (6.37 → 11.64 m^2^/g, +83%), exceeding the growth of the overall BET surface area and confirming that newly formed fine mesoporosity, rather than the pre-existing macroporous framework, accounts for most of the additional surface generated by sorption. These changes complement those established for the modified cellulose (Section 3.1.1), where an even larger relative gain in BET surface area (3.6-fold) was observed after rhenium sorption; together, the two datasets indicate that both components of the interpolymer sorption system undergo a consistent, sorption-induced refinement of their porous texture, in line with their complementary roles (anion exchange on the modified cellulose, cation exchange on the resin) within the combined system. A contribution of physically deposited inorganic salts to the observed surface-area and mass gains, rather than solely a genuine increase in mesoporosity, cannot be fully excluded from BET and TGA data alone; however, all samples were washed and dried under identical conditions before and after sorption (Section 2.8), and the disproportionate gain concentrated specifically in the fine-mesopore range, together with the persistence of the coarse macroporous background and the pronounced hysteresis development, is more consistent with genuine textural development during sorption than with simple salt crystallization on the external surface, which would be expected to reduce rather than increase the measured surface area.

### 3.2. Morphology and Elemental Composition (Sem-Eds)

#### 3.2.1. SEM-EDS of Cellulose: Pristine, Modified, and After Sorption

**Figure 6:**
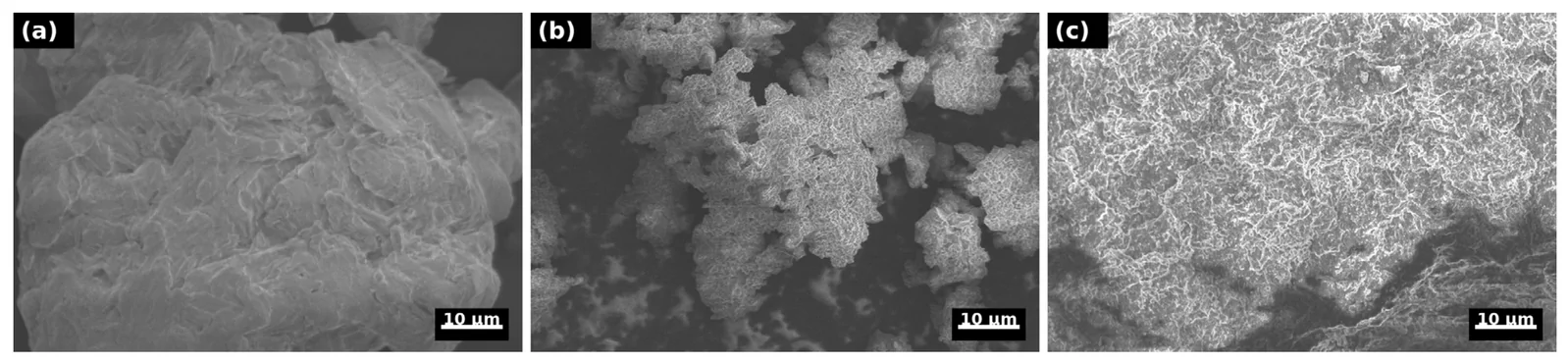
SEM images at ×5000 magnification: (**a**) pristine cellulose, (**b**) cellulose modified with PEI and cross-linked with glutaraldehyde, and (**c**) the modified sorbent after rhenium sorption from the re-extract. Accelerating voltages 1, 3 and 5 kV, respectively; SE detector; scale bars 10 µm.

The surface texture and elemental composition of pristine cellulose (Sample 1), modified Cellulose-PEI-GA (Sample 2), and the modified sorbent after rhenium sorption from the re-extract (Sample 3) were probed by SEM-EDS; acquisition conditions are summarized in Table 8. The surface morphology at ×5000 magnification (Figure 6) progresses from dense, smooth-layered pristine cellulose (a) to a loose, coral-like aggregated structure after PEI-GA functionalization (b) to a rough, network-like texture after sorption (c), consistent with the parallel gain in BET surface area across the series (1.93 → 2.42 → 8.62 m^2^/g).

Quantitative EDS compositions (Table 9) show a clear compositional progression: pristine cellulose is a simple C/O surface (30.8/69.2 at.%; O/C = 2.25, elevated relative to the theoretical cellulose value of 0.83 due to the shallow sampling depth at low accelerating voltage). Modification introduces a substantial nitrogen signal (20.8 at.%) with a corresponding drop in O/C to 0.87, confirming successful grafting of PEI and its cross-linking with glutaraldehyde.

**Figure 7:**
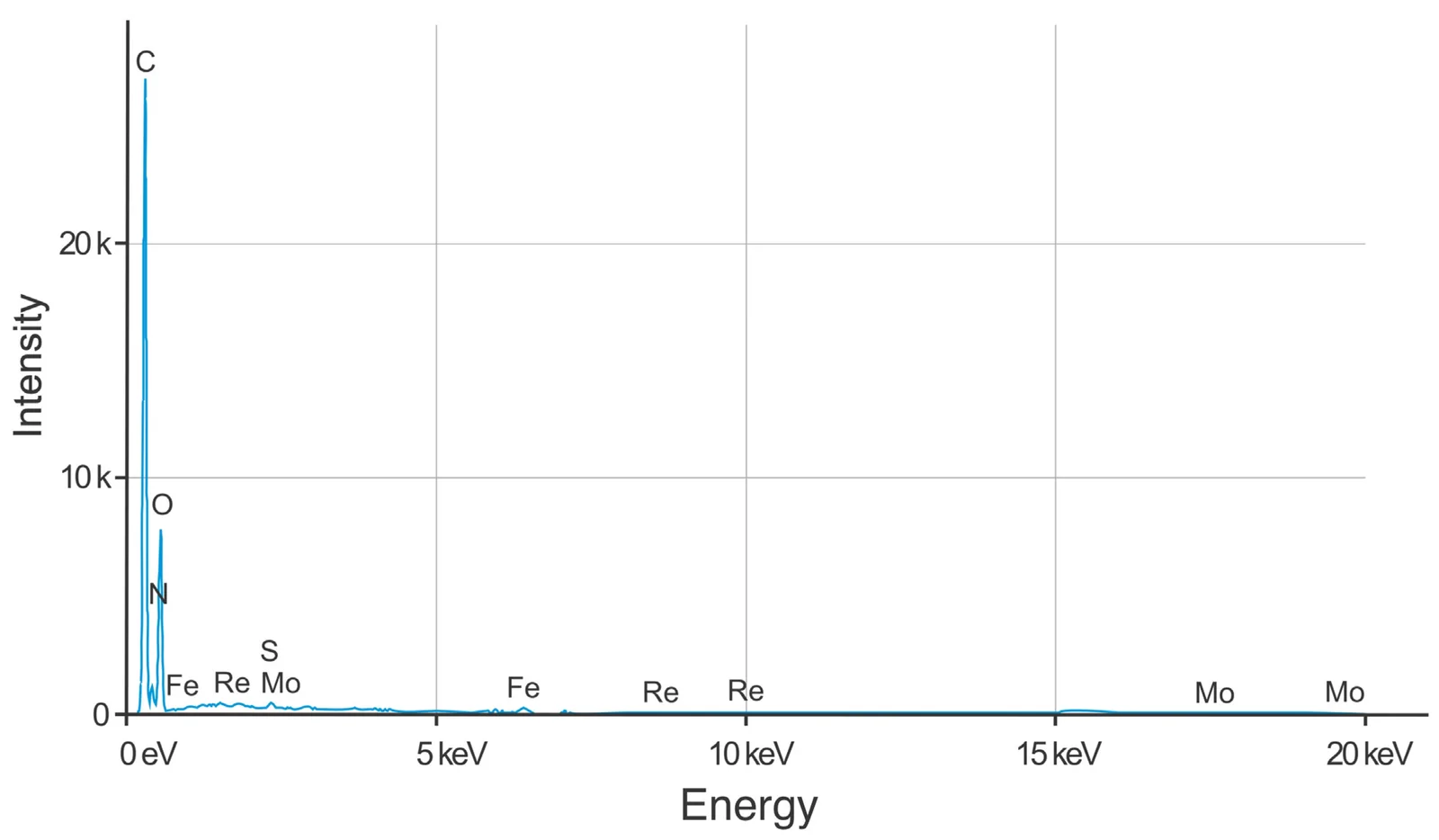
EDS spectrum of the modified cellulose after rhenium sorption from the re-extract (Sample No.3).

After sorption from the re-extract, the surface additionally carries sulfur (0.1 at.%), iron (0.3 at.%), rhenium (0.0 at.%; 0.2 wt.%), and molybdenum (0.0 at.%; 0.2 wt.%)—a combined metal loading of ≈1.6 wt.% (Figure 7)—directly confirming retention of metals and anions from the re-extract on the functionalized cellulose surface. The detection of Re and Mo at comparable atomic fractions despite their much higher atomic masses is indicative of chemisorption rather than physical trapping; iron is most probably retained through coordination with the nitrogen-bearing PEI groups, while the sulfur signal likely originates from sulfate species in the re-extract. Together, these data demonstrate the efficiency of the cellulose modification and offer direct evidence for the sorption of Fe, Mo, Re and S from the re-extract onto the functionalized matrix.

#### 3.2.2. SEM-EDS of Lewatit MonoPlus SP112H Before and After Sorption

**Figure 8:**
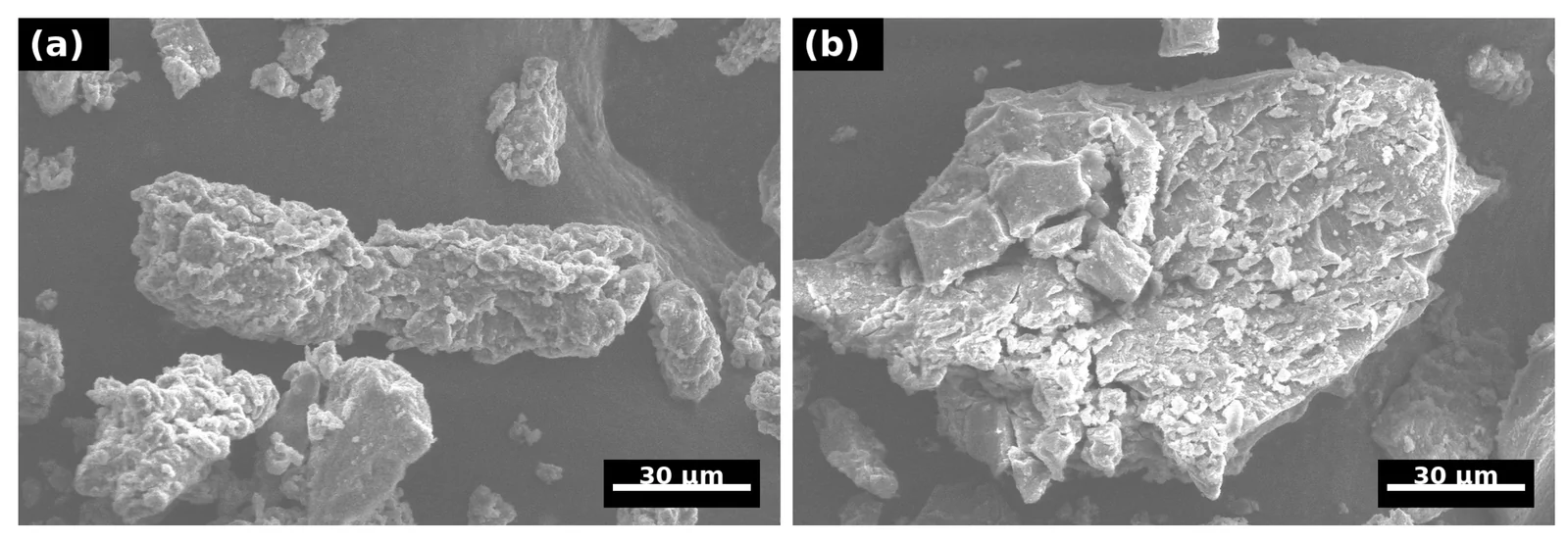
SEM images of the Lewatit MonoPlus SP112H resin (×1250): (**a**) before sorption and (**b**) after sorption. Accelerating voltage 10 kV; SE detector; scale bars 30 µm.

The second component of the interpolymer system, Lewatit MonoPlus SP112H, was examined by SEM-EDS before (Sample 4) and after (Sample 5) contact with the working solution. The surface morphology (Figure 8) changes from dense, compact granular particles before sorption to a rougher, flake-like surface with fine deposits after sorption, indicating partial surface restructuring.

The EDS composition (Table 10) is dominated by C, O, and S; the sulfur signal (6.0 → 8.8 at.%), characteristic of the sulfonic-acid (-SO_3_H) functional groups, confirms that these groups remain intact throughout sorption.

**Figure 9:**
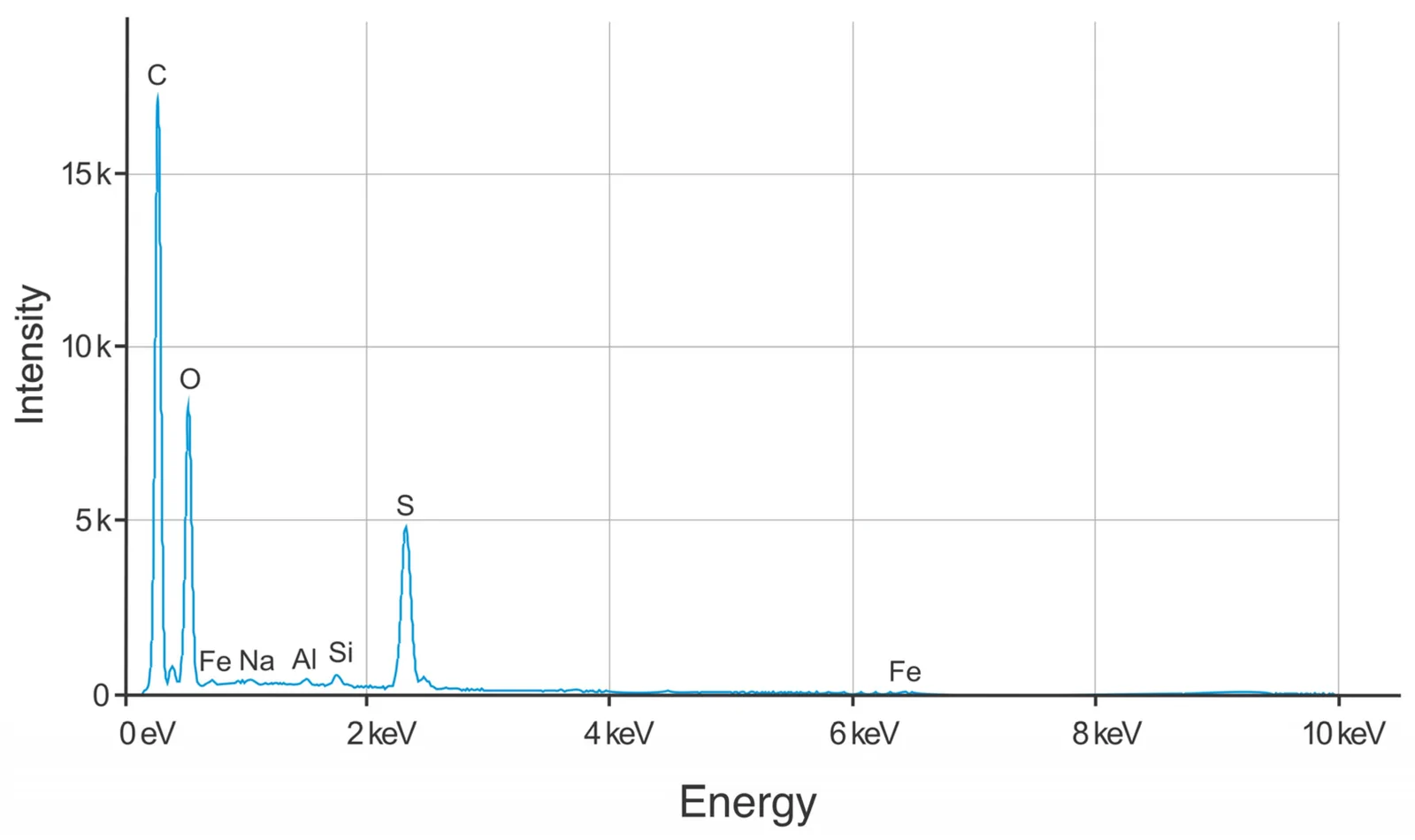
EDS spectrum of the Lewatit MonoPlus SP112H after sorption of rhenium from re-extract (Sample 5).

After sorption, the surface additionally shows iron (0.6 at.%) and trace Na, Al, and Si (≤0.4 at.%), while no rhenium was detected within the sensitivity of the method—in clear contrast to the functionalized cellulose (Sample 3), on which rhenium was clearly identified (Figure 9). This directly reflects the complementary design of the interpolymer system: the anion-exchanging cellulose (protonated PEI groups) binds anionic species including ${ReO}_{4}^{-}$, while the strong-acid resin (-SO_3_H groups) retains cationic species such as Fe. The absence of a rhenium signal on the resin is also consistent with the relatively low accelerating voltage used (10 kV), at which the Re L-line is only weakly excited; nevertheless, the result agrees fully with the anion/cation separation mechanism. Overall, the SEM-EDS results provide morphological and compositional evidence for the operating mechanism of the interpolymer system: anionic rhenium (and molybdenum) species are taken up by the anion-exchanging functionalized cellulose, while accompanying cations are retained by the cation-exchange resin, supporting the complementary roles of the two components in the selective recovery of rhenium. EDS provides only local, semi-quantitative elemental information from the analyzed spot rather than a bulk average, and the reported atomic percentages should therefore be interpreted as indicative of compositional trends rather than absolute quantification.

### 3.3. FTIR Spectroscopic Analysis

**Figure 10:**
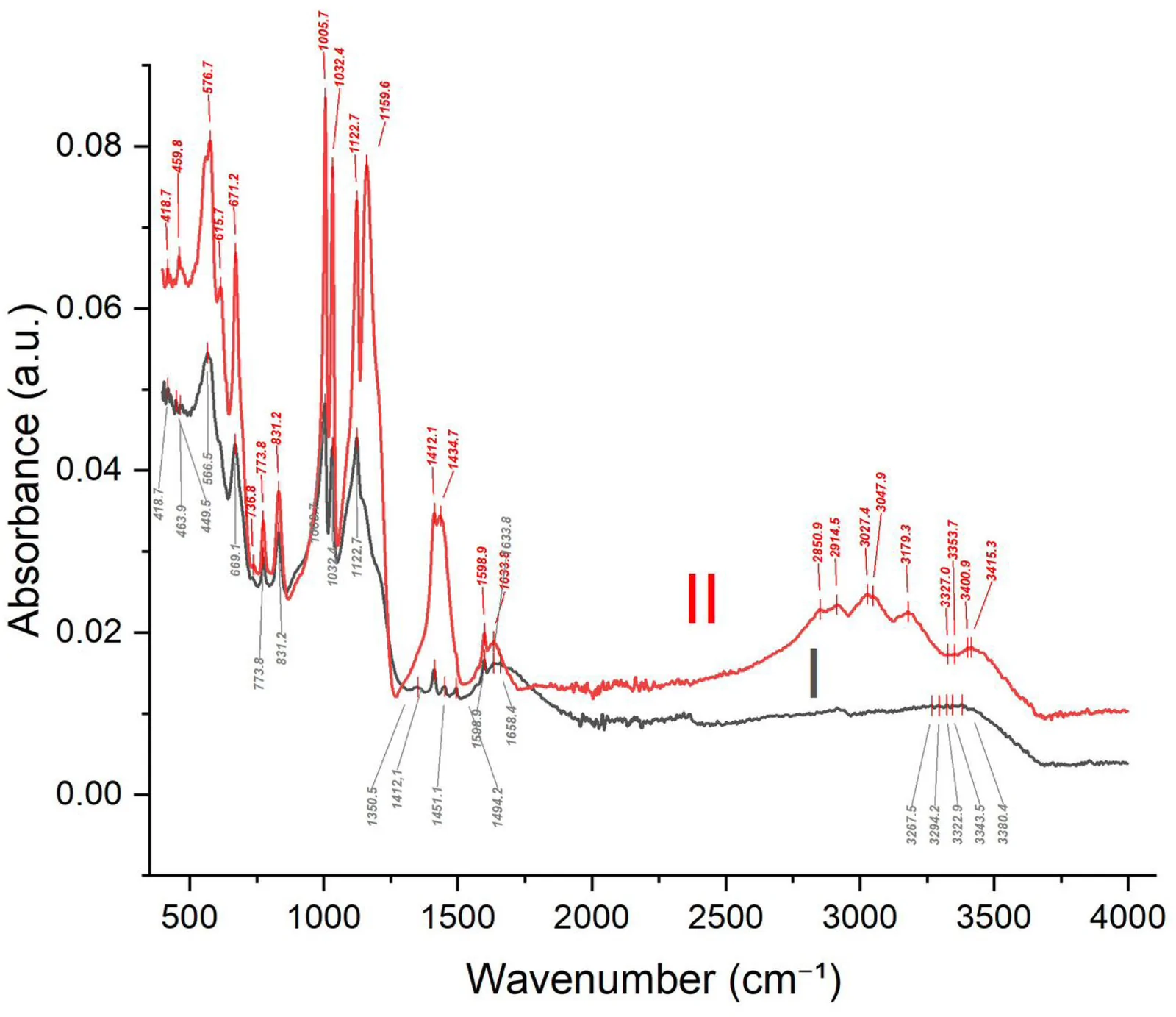
FTIR spectra of Lewatit MonoPlus SP112H before (I, grey) and after (II, red) contact with the ammoniacal re-extract.

**Figure 11:**
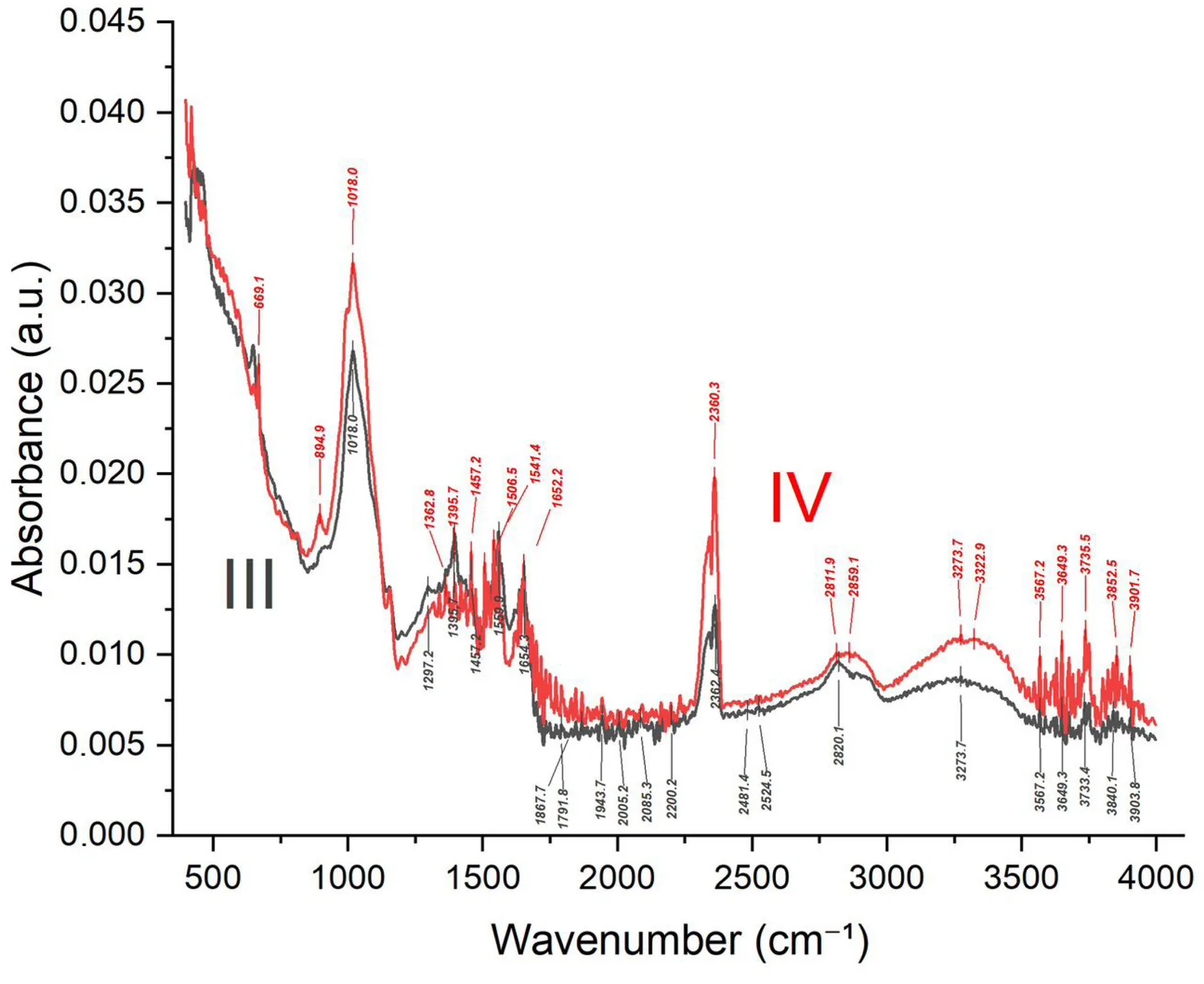
FTIR spectra of the Cellulose-PEI-GA composite before (III, grey) and after (IV, red) contact with the ammoniacal re-extract. The band at 894.9 cm^−1^ in spectrum IV is assigned to the Re=O stretching of sorbed perrhenate.

Infrared spectra were collected with a Bruker Alpha II spectrometer over 4000–400 cm^−1^ to reveal the functional groups of both interpolymer components and to track how they take part in rhenium uptake from the ammoniacal re-extract. Although molybdenum is co-sorbed alongside rhenium (Section 3.7), no FTIR band distinguishable from the Re=O stretching region (≈895 cm^−1^) or from the sulfonate/matrix bands of Lewatit could be unambiguously assigned to a Mo–O species, likely reflecting spectral overlap with the more intense neighboring bands; FTIR therefore provides direct evidence primarily for the rhenium-binding mechanism. This asymmetry is not attributable to the relative amounts sorbed, the molar uptake of Mo(VI) at the 3:3 composition is in fact substantially higher than that of Re(VII) (0.188 vs. 0.024 mmol g^−1^, from Table 11)—but to the intrinsically different spectral signatures of the two oxoanions. The Re=O asymmetric stretch of the tetrahedral ${ReO}_{4}^{-}$ ion is a single, sharp, isolated band, whereas Mo-O vibrations of molybdate/polymolybdate species are distributed over several broad, overlapping modes in the 550–950 cm^−1^ range that coincide with the already intense matrix and sulfonate bands (1018, 1032, 1122 cm^−1^), precluding their unambiguous resolution. The spectra of the strong-acid cation exchanger Lewatit MonoPlus SP112H before (I) and after (II) contact with the re-extract are compared in Figure 10, and those of the functionalized cellulose Cellulose-PEI-GA before (III) and after (IV) contact with the re-extract in Figure 11. In both figures, the weak band near 2360 cm^−1^ and the sharp features above 3500 cm^−1^ originate from atmospheric CO_2_ and uncompensated water vapor and are not related to the samples.

#### 3.3.1. Lewatit MonoPlus SP112H Before and After Sorption

To assess whether the cation-exchange component participates in rhenium binding, the functionalized resin was examined by FTIR before and after contact with the ammoniacal re-extract. Figure 10 compares the spectrum of the pristine Lewatit MonoPlus SP112H (I) with that of the resin recovered after sorption (II), allowing the matrix and sulfonic-group bands to be distinguished from any new features associated with the uptake of rhenium species. The spectrum of the pristine resin (I) is dominated by the crosslinked styrene–DVB framework and its sulfonic groups. The bands at 1032 and 1122 cm^−1^ are corresponding to the symmetric and asymmetric stretching of the -SO_3_H groups, the band at 1598 cm^−1^ to aromatic C=C ring vibrations, and the broad envelope at 3267–3380 cm^−1^ to O–H stretching of the sulfonic groups and sorbed water. The bands at 773 and 831 cm^−1^ reflect out-of-plane deformation of aromatic C-H groups, and the set below 700 cm^−1^ (418–669 cm^−1^) to skeletal vibrations of the polymer matrix.

After contact with the re-extract (II), the sulfonate and aromatic bands are retained, confirming that the resin matrix is preserved. The most pronounced change is the appearance of well-defined C-H stretching bands at 2850 and 2914 cm^−1^ and the additional bands at 1005 and 1159 cm^−1^, together with the splitting near 1412/1434 cm^−1^. No distinct band is observed in the 900–920 cm^−1^ region where the asymmetric Re=O stretching of the perrhenate ion ${ReO}_{4}^{-}$ would be expected. The absence of a perrhenate signature indicates that the cation exchanger itself does not bind ${ReO}_{4}^{-}$ to any appreciable extent, in agreement with the negligible rhenium uptake measured for the pure-resin composition [55,56,57,58,59,60].

#### 3.3.2. Cellulose-PEI-GA Before and After Sorption

The amine-functionalized cellulose is the component expected to be responsible for oxoanion binding, and it was therefore examined by FTIR under the same conditions. Figure 11 compares the spectrum of the unloaded Cellulose-PEI-GA composite (III) with that of the sorbent after contact with the ammoniacal re-extract (IV), so that bands arising from rhenium uptake can be identified against the unchanged backbone of the material. The recorded spectrum of the functionalized cellulose (III) demonstrates the expected backbone bands: the strong C-O/C-O-C stretching at 1018 cm^−1^, CH_2_ deformations near 1297 and 1457 cm^−1^, the N-H deformation of the PEI amino groups and the imine C=N of the glutaraldehyde crosslink in the 1540–1655 cm^−1^ region, and the broad O–H/N–H stretching envelope around 3273 cm^−1^. After contact with the re-extract (IV), a new, well-resolved band appears at 894.9 cm^−1^, which is absent in the spectrum of the unloaded sorbent (III). This band lies in the region characteristic of the asymmetric Re=O stretching of the perrhenate ion and is assigned to ${ReO}_{4}^{-}$ bound to the sorbent. Its appearance only on the cellulose component, and not on the Lewatit resin, provides direct spectroscopic evidence that rhenium is retained on the Cellulose-PEI-GA phase through interaction with the protonated amino groups of the PEI network. Concomitant changes in the 1360–1540 cm^−1^ region (new bands at 1362, 1395, 1506 and 1541 cm^−1^) reflect the rearrangement of the C-N and N-H vibrations of the amino groups upon binding of the oxoanion, consistent with an electrostatic/anion-exchange interaction between protonated –${NH}_{3}^{+}$ centers and ${ReO}_{4}^{-}$ [55,56,57,58,59,60].

The FTIR results support the proposed binding mechanism of the interpolymer system. The sulfonated Lewatit resin retains its matrix and sulfonic groups but shows no perrhenate signature, whereas the Cellulose-PEI-GA composite develops a distinct Re=O band at 894.9 cm^−1^ after sorption. This confirms that rhenium uptake takes place on the amine-functionalized cellulose component via its protonatable amino groups, while the cation exchanger acts mainly by maintaining the acidic local environment required for their protonation.

### 3.4. Thermal Analysis (TG-DTA-DTG)

**Figure 12:**
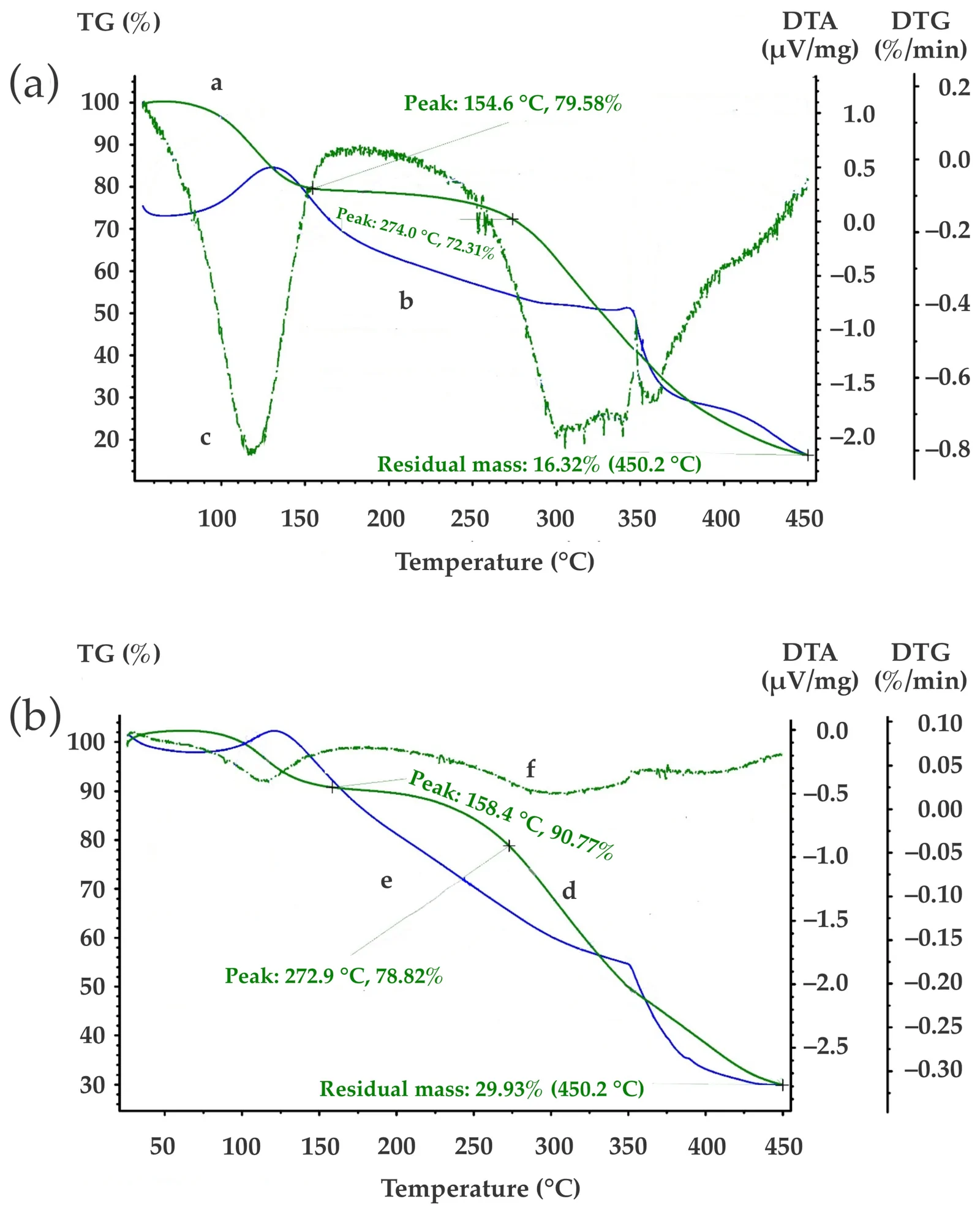
TG-DTA-DTG curves of the Cellulose-PEI-GA composite: (**a**) before sorption and (**b**) after sorption. In panel (**a**), **a**—TG (solid green line), **b**—DTA (solid blue line), and **c**—DTG (green dashed line) are shown. In panel (**b**), **d**—TG (solid green line), **e**—DTA (solid blue line), and **f**—DTG (green dashed line) are shown. The characteristic DTG peak temperatures and the residual mass at approximately 450 °C are indicated on the plots.

**Figure 13:**
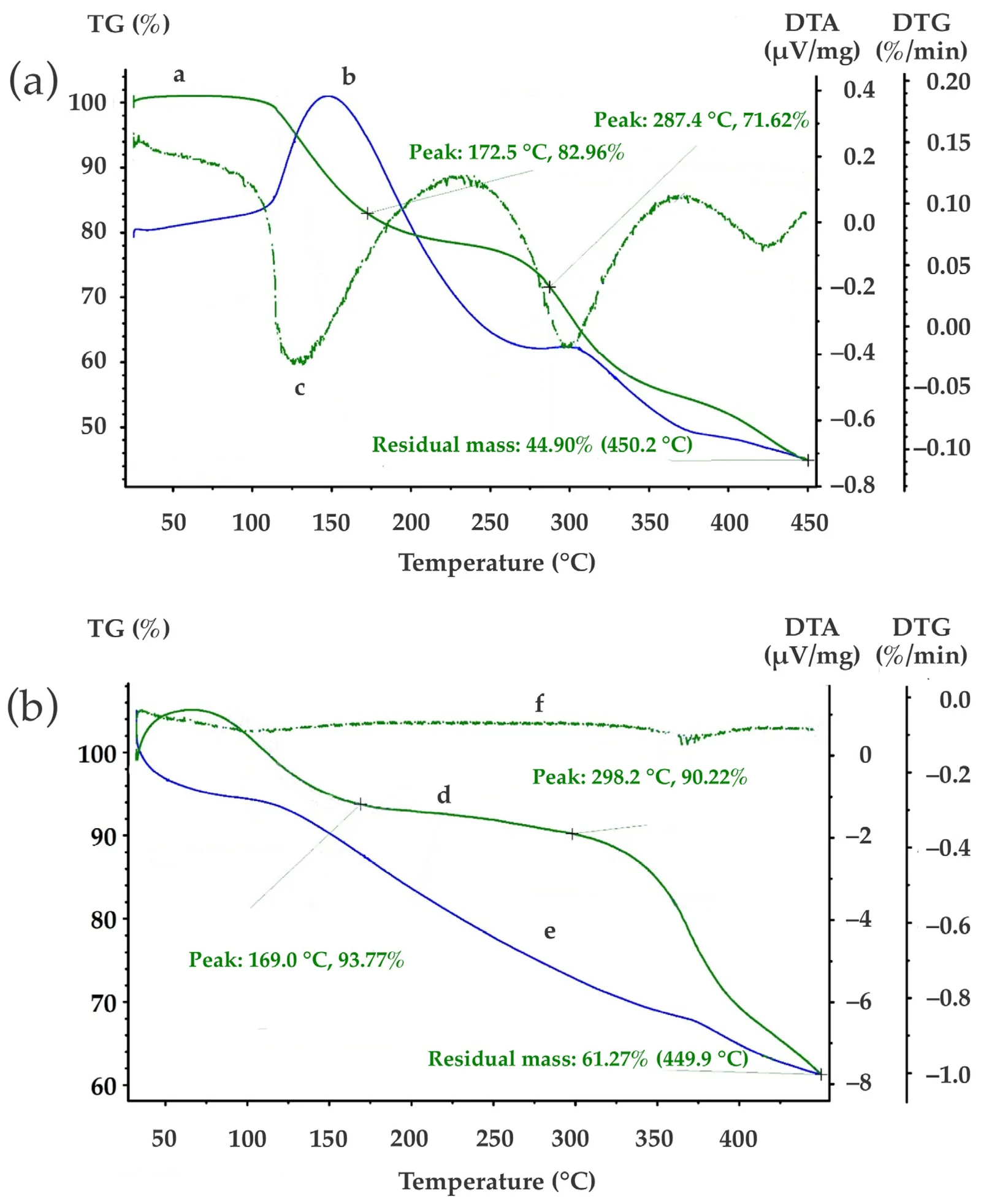
TG-DTA-DTG curves of the Lewatit MonoPlus SP112H resin: (**a**) before sorption and (**b**) after sorption. In panel (**a**), **a**—TG (solid green line), **b**—DTA (solid blue line), and **c**—DTG (green dashed line) are shown. In panel (**b**), **d**—TG (solid green line), **e**—DTA (solid blue line), and **f**—DTG (green dashed line) are shown. The characteristic DTG peak temperatures and the residual mass at approximately 450 °C are indicated on the plots.

The thermal stability of the two interpolymer system components was studied by simultaneous thermogravimetry (TG), differential thermal analysis (DTA) and derivative thermogravimetry (DTG). Figure 12 shows the curves of the Cellulose-PEI-GA composite before (a) and after (b) sorption, and in Figure 13 those of the Lewatit MonoPlus SP112H resin before (a) and after (b) sorption are shown. Each panel presents the TG (green), DTA (blue) and DTG (dashed green) traces together, with the main DTG peak temperatures and the residual mass at about 450 °C marked on the plots.

#### 3.4.1. Cellulose-PEI-GA

Before sorption (Figure 12a), the composite undergoes thermal decomposition in two main stages. The first stage is characterized by a maximum DTG at 154.6 °C, corresponding mainly to the removal of bound water and the initial degradation of the polymer network. The second decomposition stage, with a DTG maximum at 274.0 °C, is associated with the degradation of the cellulose backbone together with the polyethyleneimine chains and the glutaraldehyde-crosslinked structure. At 450 °C, the residual mass is 16.32%. After sorption (Figure 12b), the thermal decomposition pattern remains similar. The characteristic DTG maxima are observed at 158.4 °C and 272.9 °C, indicating that sorption has only a minor effect on the decomposition temperatures of the composite. However, the residual mass increases to 29.93% at 450 °C. The higher residual mass is attributed to the inorganic species retained by the composite during sorption, which remain as thermally stable residues after heating.

#### 3.4.2. Lewatit MonoPlus SP112H

Before sorption (Figure 13a), the resin exhibits two main stages of thermal decomposition. The first stage, with a DTG maximum at 172.5 °C, is attributed mainly to the removal of bound water associated with the sulfonic acid groups. The second stage, with a DTG maximum at 287.4 °C, corresponds to the desulfonation of the resin and the degradation of the styrene–divinylbenzene polymer matrix. At 450 °C, the residual mass is 44.90%. After sorption (Figure 13b), the thermal decomposition pattern remains generally unchanged. The DTG characteristic peaks are observed at 169.0 °C and 298.2 °C that indicate the thermal stability of resin framework after sorption. At 450 °C, residual mass increases to 61.27%. The higher residual mass is attributed to inorganic species retained by the resin during sorption, which remain as thermally stable residues after heating.

For both sorbents, the thermal decomposition behavior changes only slightly after sorption, whereas the residual mass increases significantly. For the Cellulose-PEI-GA composite, the residual mass increases from 16.32% to 29.93%, while for the Lewatit MonoPlus SP112H resin it increases from 44.90% to 61.27%. These results provide additional evidence for the uptake of inorganic species by both sorbents and are consistent with the FTIR and SEM–EDS analyses. Because sample preparation (washing, drying) was identical before and after sorption, the higher residual mass is attributed primarily to retained inorganic sorbate species rather than to differences in sample handling.

### 3.5. Effect of the Cation-to-Anion Exchanger Ratio

**Figure 14:**
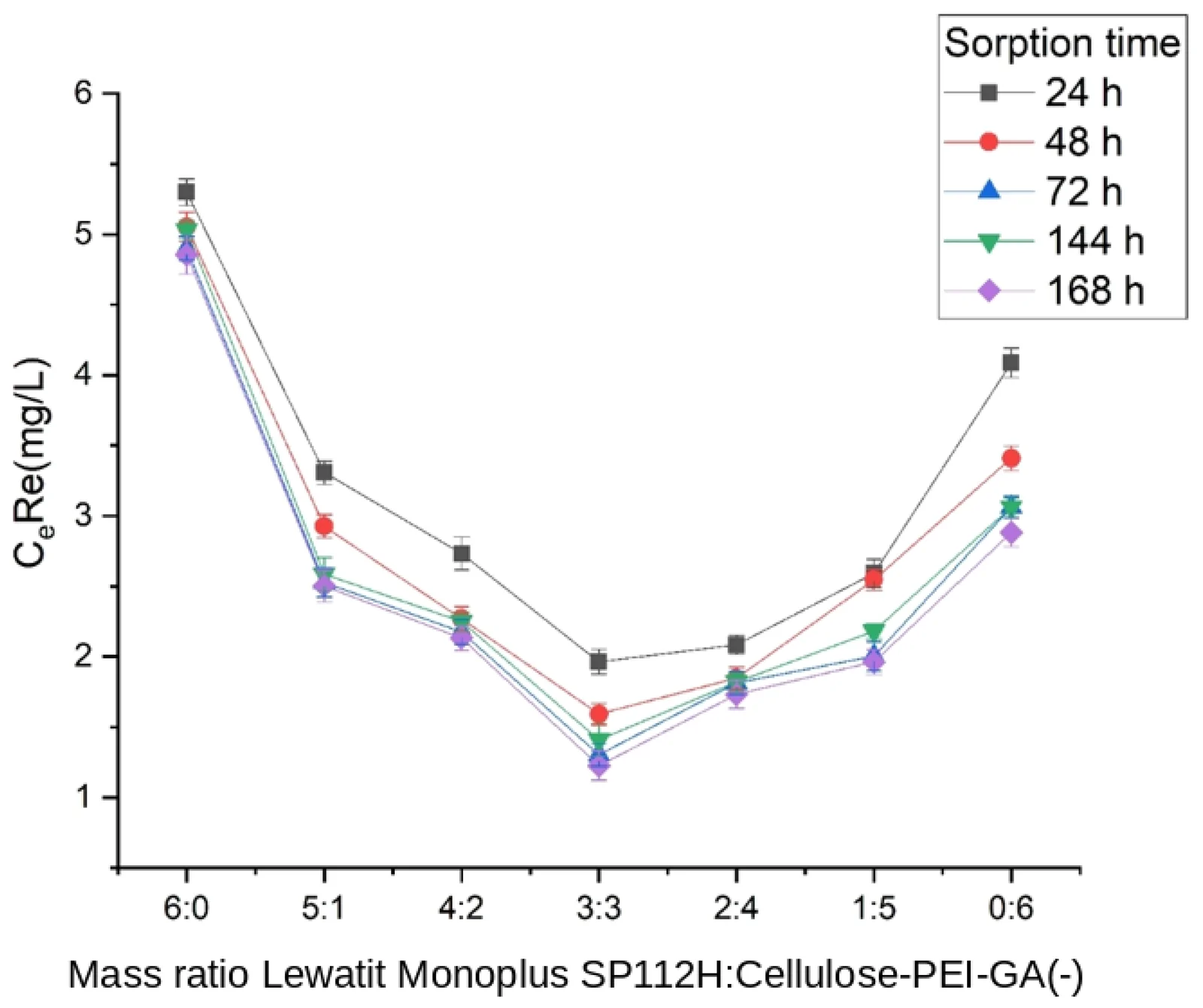
Residual Re(VII) concentration as a function of the Lewatit MonoPlus SP112H: Cellulose-PEI-GA mass ratio for various contact times (24–168 h). The ratio is expressed as mass parts of a fixed total sorbent loading of 1.0 g (e.g., 3:3 corresponds to 0.5 g: 0.5 g). The minimum at the 3:3 ratio corresponds to the maximum rhenium uptake. Data points correspond to the average of three independent runs; error bars represent the standard deviation (mean ± SD, *n* = 3), with SD below 2%.

**Figure 15:**
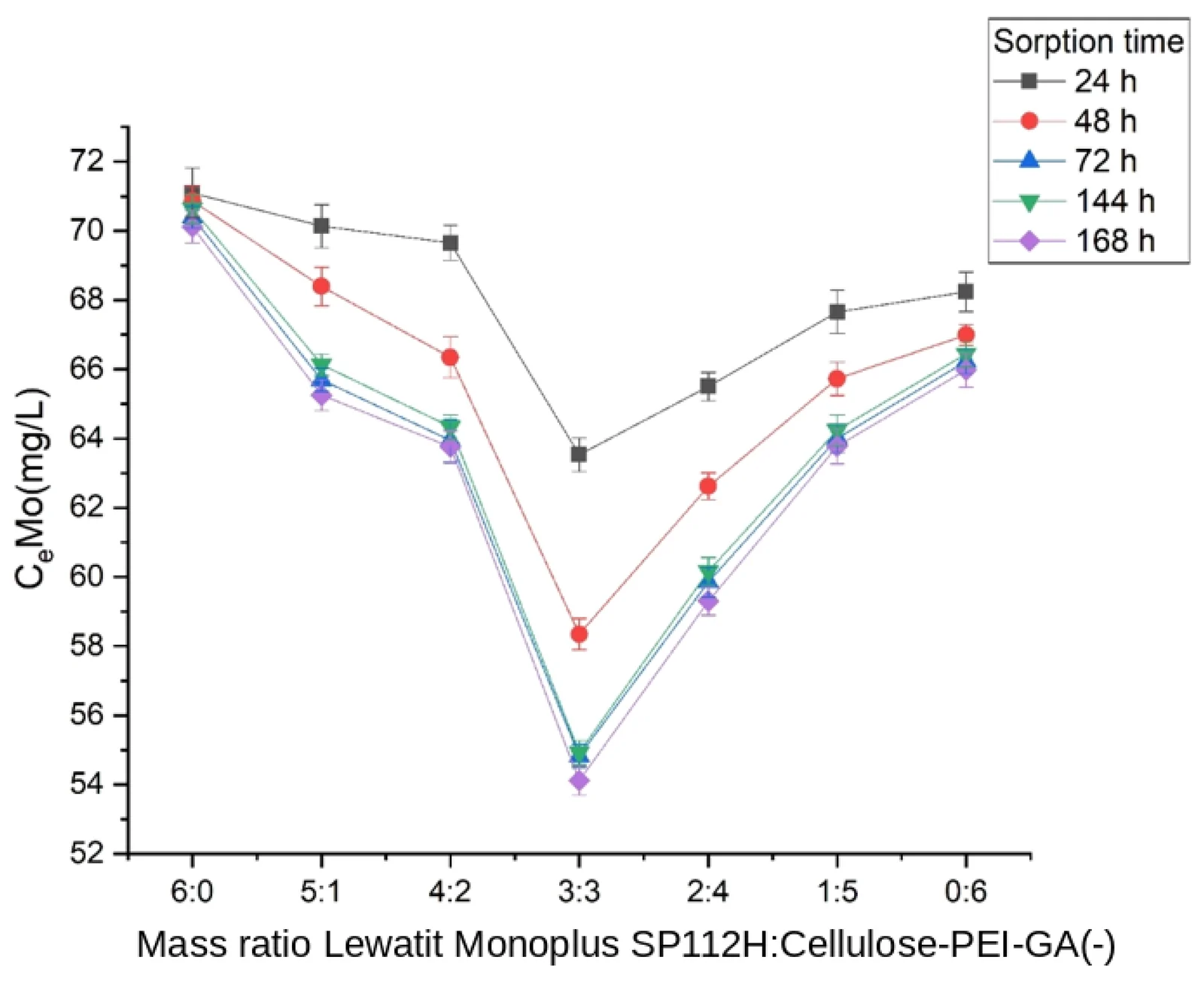
Residual Mo(VI) concentration as a function of the Lewatit MonoPlus SP112H: Cellulose-PEI-GA mass ratio for various contact times (24–168 h). The minimum at the 3:3 ratio corresponds to the maximum molybdenum uptake. Data points correspond to the average of three independent runs; error bars represent the standard deviation (mean ± SD, *n* = 3), with SD below 2%.

In addition to the target rhenium, molybdenum was monitored in the same sorption experiments as the principal co-occurring oxoanion, in order to assess the Re/Mo selectivity of the interpolymer system under identical conditions. The uptake of Re(VII) and Mo(VI) from the ammoniacal re-extract was studied for seven Lewatit MonoPlus SP112H: Cellulose-PEI-GA ratios, from the pure cation exchanger (6:0) to the pure anion-binding composite (0:6). The residual metal content plotted against composition and contact time is given in Figure 14 and Figure 15 for Re and Mo, respectively. For both metals, the residual concentration passes through a minimum at the 3:3 ratio, i.e., the sorption reaches a maximum when the cation and anion exchangers are present in equal mass proportions. This extremum is characteristic of interpolymer systems and reflects the mutual activation of the two polymer phases: the strongly acidic sulfonic groups of Lewatit maintain a low local pH that promotes protonation of the PEI amino groups, which in turn bind the ${ReO}_{4}^{-}$ and, under the alkaline conditions of the ammoniacal re-extract (pH 11.98) used here, the simple molybdate ion Mo$O_{42}^{-}$ rather than the polymolybdate species that dominate only at pH < 4 (Supplementary Table S2). At the extreme compositions, the effect weakens—the pure cation exchanger (6:0) shows almost no oxoanion uptake, confirming that anion binding takes place on the cellulose-PEI phase, whereas the pure composite (0:6) lacks the acidifying contribution of the resin. The non-monotonic behavior between 72 and 144 h reflects a partial redistribution of oxoanions between the phases and experimental scatter near equilibrium.

### 3.6. Recovery and Distribution Coefficients

Equilibrium parameters were evaluated at 168 h. For the optimal 3:3 composition, the rhenium recovery reached 77.7% with a distribution coefficient Kd = 3678 mL g^−1^, and the corresponding uptake was 4.51 mg g^−1^. Molybdenum was extracted to a markedly lower degree under the same conditions (24.0%, Kd = 333 mL g^−1^). Both metals reach their highest Kd at the 3:3 ratio, as shown in Figure 14 (Table 11). To evaluate whether the performance of the 3:3 composition reflects a cooperative effect rather than a simple additive contribution of the two components, the expected uptake was estimated from the individual capacities of the pure sorbents (compositions 6:0 and 0:6 in Table 11), weighted by their respective mass fractions in the 3:3 system (0.5 g each): q_add_ = 0.5 × q(0:6) + 0.5 × q(6:0) = 0.5 × 2.76 + 0.5 × 0.67 = 1.72 mg g^−1^. The experimentally observed uptake at the 3:3 composition (4.51 mg g^−1^) exceeds this additive estimate by a factor of ~2.6, indicating that the enhanced performance of the interpolymer system cannot be explained by a simple combination of independent contributions and supports the proposed cooperative mechanism. Spatial separation of the two components into permeable bags is not merely an experimental convenience but the defining feature of an interpolymer system as such [27,28,29,30,31,32]: the two ion-exchange phases interact indirectly through the shared solution rather than through direct physical contact. A directly mixed (unbagged) configuration would therefore constitute a fundamentally different object of study—a physical sorbent blend rather than an interpolymer system—and was accordingly outside the scope of the present work.

The 3:3 composition was selected as optimal with respect to rhenium recovery, uptake capacity, and distribution coefficient—the primary objective of this study—rather than with respect to the Re/Mo separation factor β, which in fact reaches its highest value at the 1:5 composition (β = 15.54, Table 11) despite its lower absolute Re recovery (64.3%) and uptake (3.74 mg g^−1^). Recovery/capacity and selectivity therefore respond differently to the Lewatit MonoPlus SP112H: Cellulose-PEI-GA ratio and should not be conflated: compositions richer in the anion-exchanging cellulose component favor higher Re uptake, whereas a moderate excess of the cation-exchange resin (1:5) favors Re/Mo discrimination. The 3:3 ratio was adopted throughout the remainder of this work because maximizing rhenium recovery, rather than the Re/Mo separation factor per se, was the primary practical objective, given that downstream purification of the loaded eluate can further address residual molybdenum.

### 3.7. Re/Mo Selectivity

**Figure 16:**
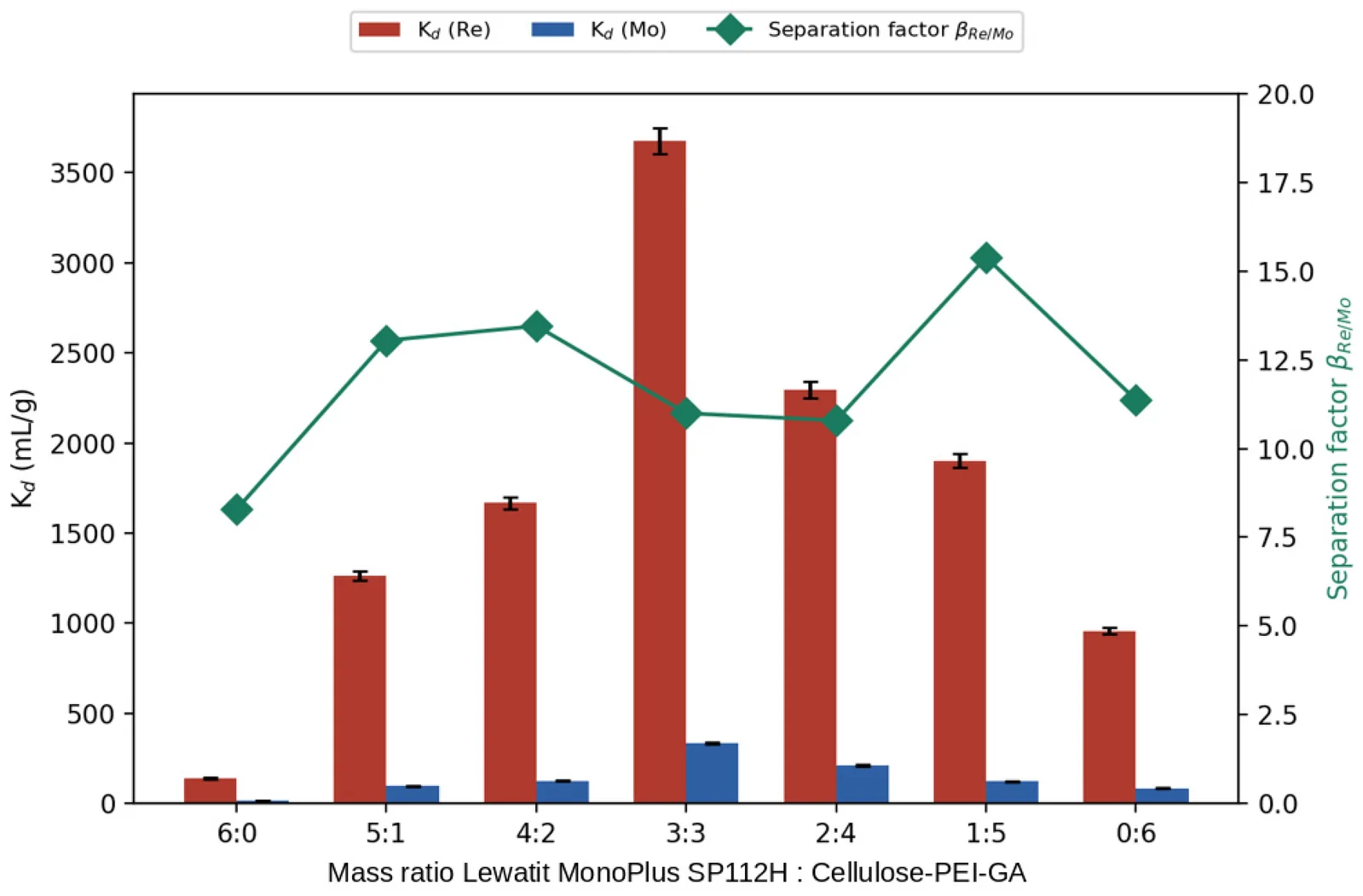
Distribution coefficients K_d_ of Re(VII) and Mo(VI) (bars, left axis) and the Re/Mo separation factor β (line, right axis) for the studied interpolymer compositions at 168 h. Bars show mean values from three independent experiments (mean ± SD, *n* = 3), with SD below 2%.

Because both metals are sorbed from the same solution, the ratio of their distribution coefficients gives a true separation factor, β(Re/Mo) = Kd(Re)/Kd(Mo). Across all compositions β ranged from 8.8 to 15.5, i.e., the system preferentially binds rhenium over molybdenum by roughly an order of magnitude (Figure 16). This preference follows from the lower surface charge density and weaker hydration of the singly charged ReO4^−^ relative to the doubly charged molybdate, which favors its interaction with the protonated amino groups. It should be emphasized that the selectivity of the proposed process is not governed by the sorbent alone. The overall hydrometallurgical scheme provides selectivity at the process level: the preceding solvent-extraction stage (trialkylamine-2–ethylhexanol–kerosene followed by ammonia stripping) removes the bulk of the competing cations and delivers a re-extract from which the interpolymer system then concentrates rhenium with a high Re/Mo separation factor. Sorbent-level and process-level selectivity therefore act in series, which is the practical basis for selective rhenium recovery from the complex Zhezkazgan liquors. This division of labor can be quantified directly from the process data: comparing the Re/Mo ratio in the industrial feed (22.73/143.26 = 0.159) with that in the re-extract used for sorption (5.494/71.23 = 0.077) gives an extraction-stage separation factor of only ~0.49—indicating that the solvent-extraction step does not itself fractionate Re from Mo (and, if anything, slightly favors Mo retention), consistent with its intended role of removing other competing cations (Fe, Al, Si, and sulfate) rather than achieving Re/Mo selectivity. The full Re/Mo separation factor (β = 8.8–15.5) is therefore attributable almost entirely to the interpolymer system sorption stage.

### 3.8. Sorption Kinetics

**Figure 17:**
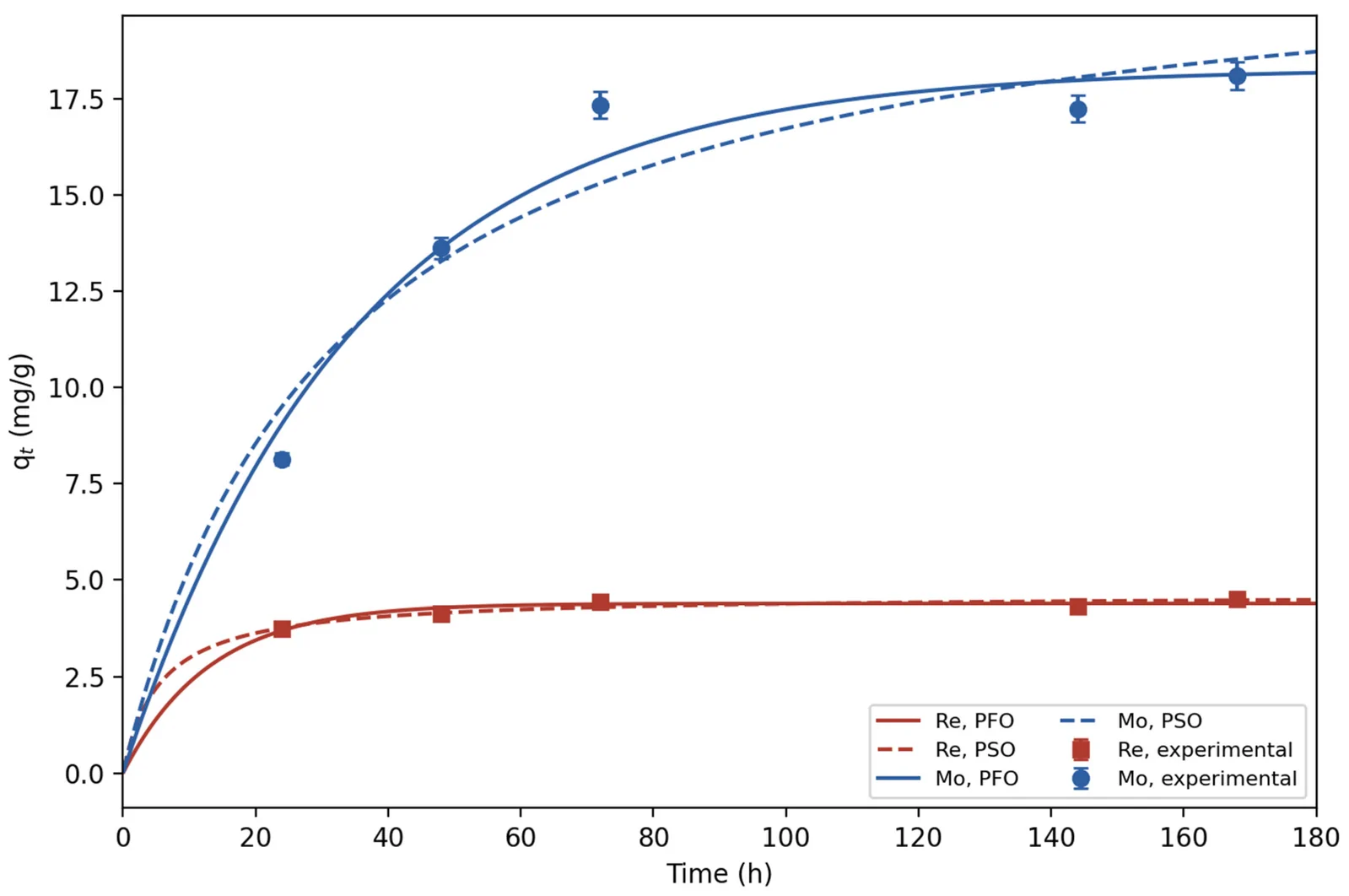
Sorption kinetics of Re(VII) and Mo(VI) on the 3:3 interpolymer system: experimental points (mean ± SD, *n* = 3, SD ≤ 2%) and pseudo-first-order (solid lines) and pseudo-second-order (dashed lines) model fits.

The kinetics of Re and Mo uptake by the optimal 3:3 composition are shown in Figure 17. The uptake is fast during the first 24–48 h and approaches a plateau by 72 h. Four kinetic and diffusion models were applied to the experimental data: pseudo-first-order (PFO) and pseudo-second-order (PSO) rate laws, the Weber–Morris intraparticle diffusion model, the Elovich chemisorption model, and the Boyd film/particle-diffusion model.

#### 3.8.1. Pseudo-First-Order (PFO) and Pseudo-Second-Order (PSO) Models

The experimental uptake curves together with the PFO (solid lines) and PSO (dashed lines) model fits are shown in Figure 17. For rhenium both models gave a comparable description (R^2^ = 0.875 for PFO and 0.898 for PSO), with calculated q_e_ values (4.39 and 4.62 mg g^−1^) close to the experimental one (4.51 mg g^−1^). For molybdenum the PFO model was superior (R^2^ = 0.951 vs. 0.898) with calculated qe values (18.22 and 21.96 mg g^−1^) compared with the experimental value (18.03 mg g^−1^). The closeness of the two fits indicates that no single reaction-order model is uniquely preferred, consistent with a mixed rate control. The full set of kinetic constants for all compositions is given in Table 12 and Table 13.

#### 3.8.2. Weber–Morris Intraparticle Diffusion

**Figure 18:**
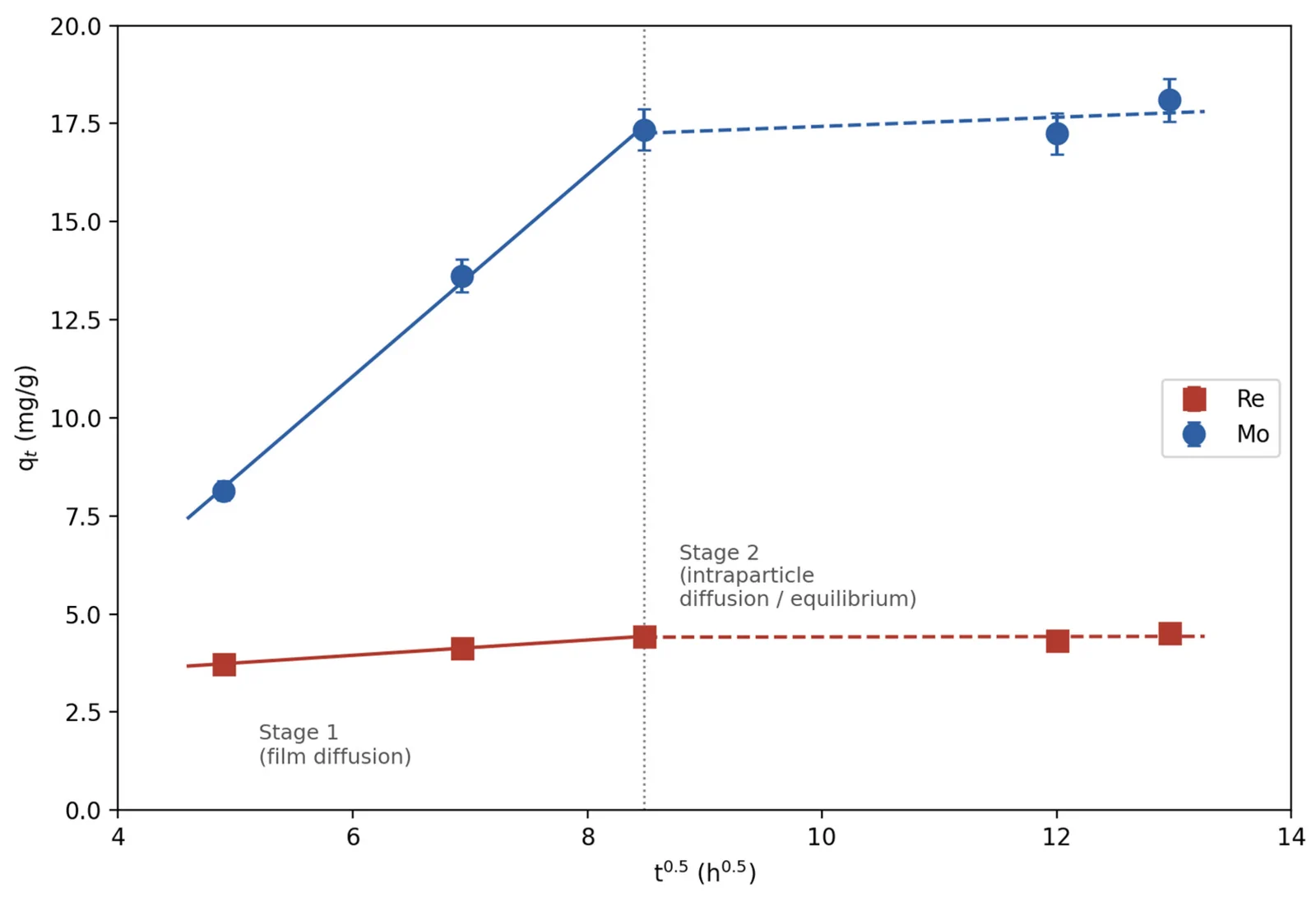
Weber–Morris intraparticle-diffusion plots (qt vs. t0.5) for Re(VII) and Mo(VI) sorption on the 3:3 interpolymer composition, resolved into two linear stages (solid: 24–72 h film-diffusion stage; dashed: 72–168 h intraparticle-diffusion/equilibrium stage). Points are mean ± SD (*n* = 3, SD ≤ 3%).

To identify the rate-controlling step, the data were further examined with the Weber–Morris intraparticle diffusion-based model (Figure 18). The qt vs. t^0.5^ plot was not linear over the whole-time range for either metal but resolved into two distinct linear stages, separated at t ≈ 72 h: a steep initial stage (24–72 h) followed by a much flatter stage (72–168 h) close to equilibrium. For rhenium, the first-stage slope (kid,1 = 0.195 mg·g^−1^·h-0.5) was roughly 45 times steeper than the second-stage slope (kid,2 = 0.0044 mg·g^−1^·h-0.5), with intercepts C1 = 2.78 mg·g^−1^ and C2 = 4.37 mg·g^−1^. For molybdenum the contrast was even sharper (kid,1 = 2.57 vs. kid,2 = 0.116 mg·g^−1^·h-0.5), and the first-stage intercept was negative (C1 = −4.38 mg·g^−1^), indicating that a substantial fraction of the eventual Mo uptake occurred within the first sampling interval (before 24 h), faster than the first linear segment alone would predict—consistent with rapid initial film-diffusion-controlled binding at the most accessible surface sites. In both cases, neither line passes through the origin, and the change in slope between the two stages is consistent with intraparticle diffusion not being the sole rate-controlling step: an initial, faster stage dominated by external (film) diffusion and boundary-layer transport across the particle surface is followed by a much slower stage governed by diffusion within the particle and approach to equilibrium [61,62]. This is consistent with the spatially separated configuration of the interpolymer pair, in which oxoanion transport between the two polymer domains is mediated by the surrounding electrolyte.

#### 3.8.3. Elovich Model

**Figure 19:**
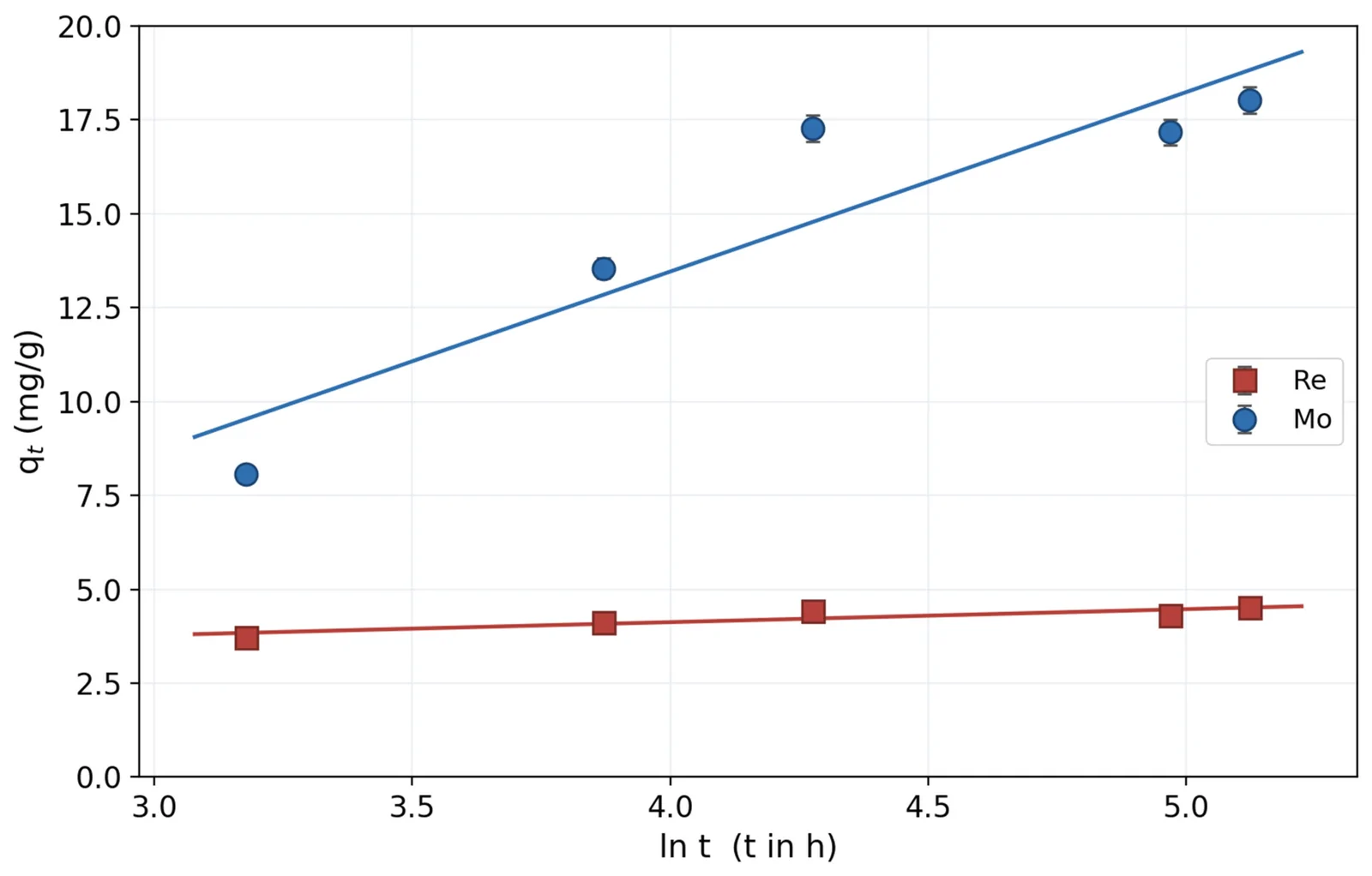
Elovich-model plots (q_t_ vs. ln t) for Re(VII) and Mo(VI) sorption on the 3:3 interpolymer composition. Solid lines are fits to the linearized Elovich equation, q_t_ = (1/β)ln(αβ) + (1/β)ln(t); α(Re) = 951 mg·g^−1^·h^−1^, β(Re) = 2.889 g mg^−1^ for rhenium, and α(Mo) = 1.465 mg·g^−1^·h^−1^, β(Mo) ≈ 0.211 g mg^−1^ for molybdenum. Points are mean ± SD (*n* = 3, SD ≤ 2%).

The Elovich equation, which assumes a set of discrete active sites with a linearly increasing activation energy for chemisorption, was applied to the uptake data in its linearized form, qt = (1/β)ln(αβ) + (1/β)ln(t) (Figure 19). For rhenium the fit gave an initial sorption rate α(Re) = 951 mg·g^−1^·h^−1^ and a desorption constant β(Re) = 2.889 g mg^−1^, consistent with a fast initial uptake followed by a rapidly decreasing rate as available sites are occupied. For molybdenum, the fit gave α(Mo) = 1.465 mg·g^−1^·h^−1^ and β(Mo) ≈ 0.211 g mg^−1^, i.e., a markedly slower initial rate but a lower β, consistent with the lower overall affinity of the interpolymer system for molybdate relative to perrhenate under these conditions. The much higher α(Re)/α(Mo) ratio (≈650) mirrors the same Re-Over-Mo preference already evident from the recovery and Kd data (Table 12 and Table 13).

#### 3.8.4. Boyd Model

**Figure 20:**
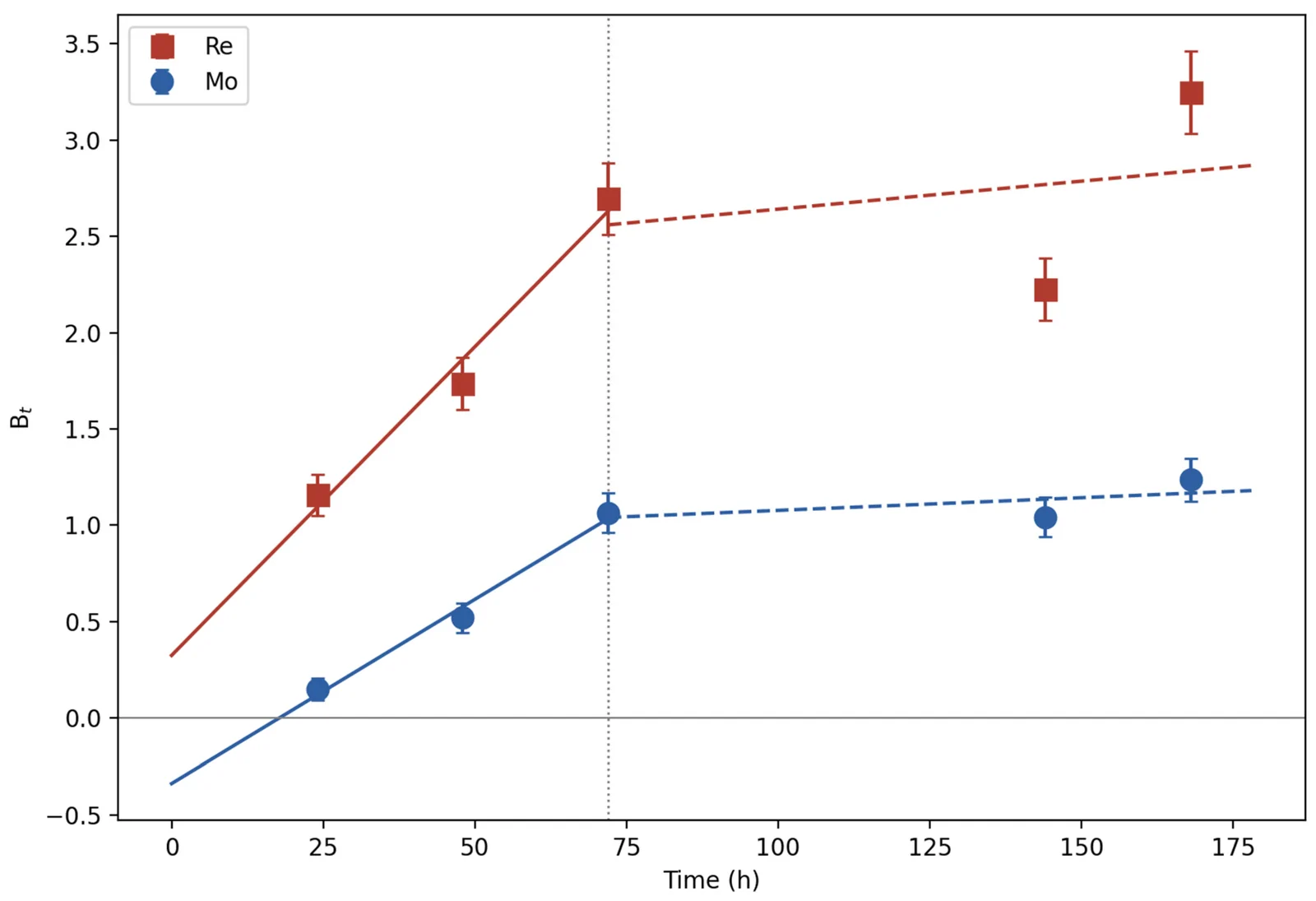
Boyd plots (Bt vs. contact time) for Re(VII) and Mo(VI) sorption on the 3:3 interpolymer composition, resolved into two linear stages with a breakpoint at t ≈ 72 h (solid: film-diffusion-dominated stage; dashed: intraparticle-diffusion/equilibrium stage). Points are mean ± SD (*n* = 3, SD ≤ 2%).

To further discriminate between film diffusion and intraparticle diffusion as the rate-controlling step, the Boyd model was applied using the Reichenberg approximation to convert the fractional uptake F = q_t_/q_e_ into the mathematical function Bt at each contact time. As with the Weber–Morris’s analysis, the Bt-t plot (Figure 20) was better described by two linear segments than by a single line over the whole-time range, with the same breakpoint at t ≈ 72 h. In the first stage (24–72 h), B1(Re) = 0.0320 h^−1^ and B1(Mo) = 0.0191 h^−1^, corresponding to effective diffusion coefficients Di,1(Re) ≈ 2.0 × 10–14 m^2^ s^−1^ and Di,1(Mo) ≈ 1.2 × 10–14 m^2^ s^−1^. In the second stage (72–168 h), the slopes dropped roughly an order of magnitude, B2(Re) = 0.0029 h^−1^ and B2(Mo) = 0.0013 h^−1^ (Di,2(Re) ≈ 1.8 × 10–15 m^2^ s^−1^, Di,2(Mo) ≈ 8.3 × 10–16 m^2^ s^−1^). Neither stage passes through the origin (intercepts of 0.33/−0.34 for Re/Mo in stage 1, and 2.35/0.95 in stage 2), supporting, in agreement with the Weber–Morris analysis, the involvement of both film and intraparticle diffusion, with the relative contribution of intraparticle (particle-internal) diffusion becoming progressively more limiting as the system approaches equilibrium after ~72 h.

It should be emphasized that the PFO, PSO, Weber–Morris, Elovich, and Boyd models are empirical descriptions of the overall uptake curve and cannot, on their own, unambiguously identify the molecular-level rate-controlling step in a multi-stage ion-exchange process (external film diffusion, ion-exchange reaction, intraparticle diffusion, and site competition). The comparable fit quality of PFO and PSO, and the two-stage behavior common to both the Weber–Morris and Boyd analyses, are therefore interpreted as indicative of a diffusion-influenced, mixed-control process rather than as direct proof of a specific mechanism; confirming the individual contribution of each step would require complementary approaches (e.g., particle-size-dependence studies or temperature-variable kinetics) beyond the scope of the present work.

### 3.9. Desorption of Rhenium

**Figure 21:**
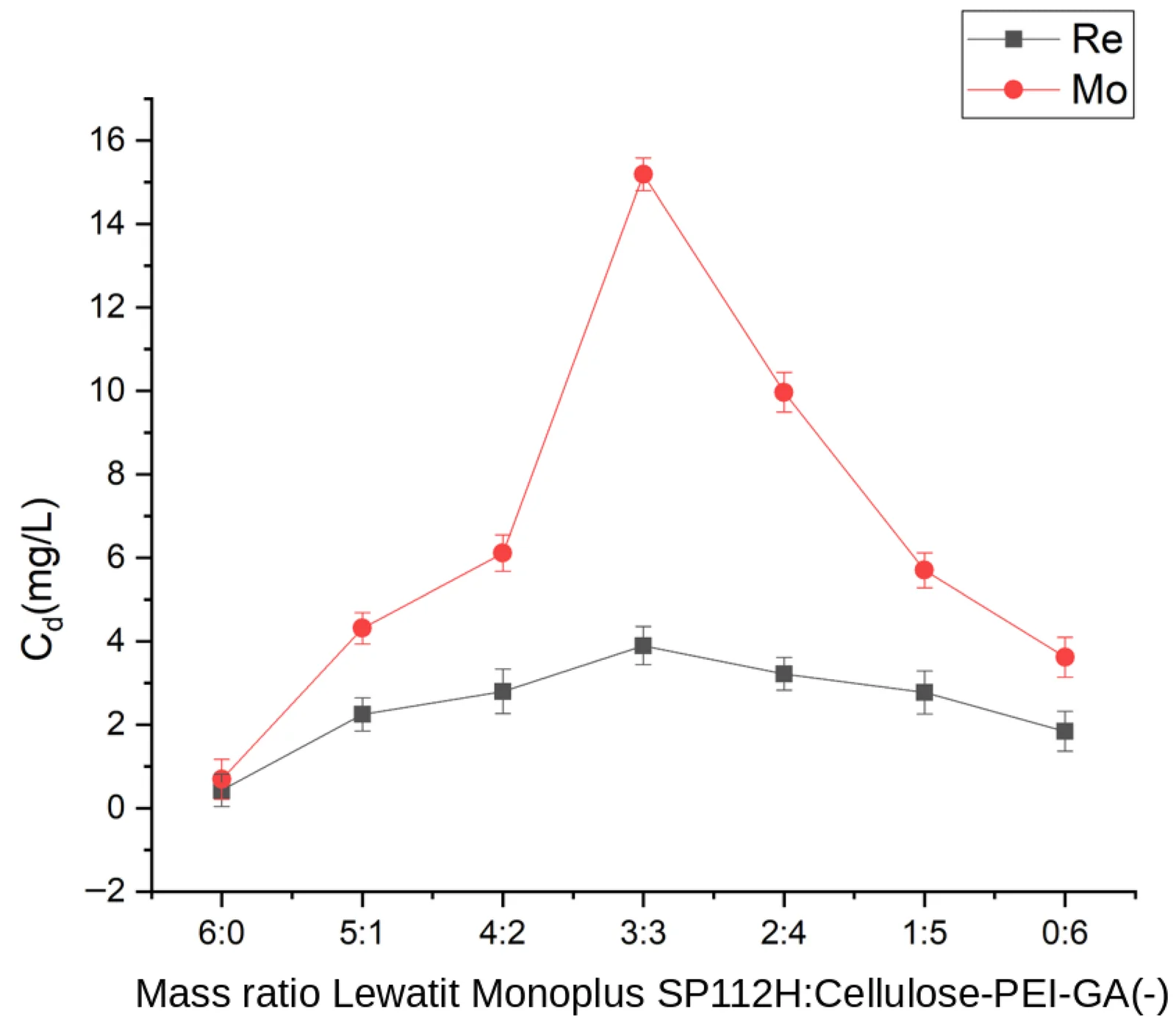
Desorption degree of Re(VII) and Mo(VI) from the interpolymer systems in 0.5 M HCl as a function of the Lewatit MonoPlus SP112H: Cellulose-PEI-GA mass ratio (24 h, room temperature). Bars show mean values (*n* = 3, mean ± SD).

The desorption behavior of rhenium and molybdenum across the studied compositions is presented in Figure 21 (numerical values are summarized in Table 14). The desorption efficiency increased with increasing interaction between the acidic and basic polymer components, reaching a maximum for the equal-mass composition (3:3). Under these conditions, the desorption efficiencies reached 91.4% for rhenium and 89.1% for molybdenum. A further increase in the Cellulose-PEI-GA content resulted in a gradual decrease in desorption efficiency. The lowest values were observed for the individual polymers, confirming that cooperative interactions between the acidic and basic functional groups not only enhance sorption but also facilitate efficient regeneration of the interpolymer system. The high desorption efficiencies obtained for the 3:3 composition indicate that the sorbed metal species are strongly retained during sorption while remaining readily recoverable during the regeneration stage, demonstrating the good reusability of the interpolymer system.

### 3.10. Comparison with Previous Studies

To place the performance of the proposed interpolymer system in context, Table 15 compares it with anion-exchange resins and interpolymer sorbents reported for rhenium and molybdenum recovery. A direct comparison of absolute capacities must be made with caution, because the reported maxima are mostly Langmuir capacities (qmax) obtained from model solutions at high initial concentrations and full saturation, whereas the present values were obtained from a real ammoniacal re-extract of fixed and comparatively low rhenium concentration (C_0_ ≈ 25 mg L^−1^), referred to the total mass of the interpolymer pair. Under such conditions, the uptake is limited by the amount of metal available in solution rather than by the intrinsic capacity of the sorbent, so the values in the last row of Table 15 represent operating capacities for a realistic feed rather than saturation maxima.

Commercial and tailor-made resins reach high saturation capacities for ${ReO}_{4}^{-}$, for example 707 and 446 mg g^−1^ for the bifunctional Purolite A530E/A532E, 437 mg g^−1^ for D318, or up to 435 mg g^−1^ for recently developed amine resins. For MoO_4_^2−^, capacities up to 414–464 mg g^−1^ have been reported for modified D301 materials. These figures are obtained at metal concentrations higher by several orders of magnitude than those of the re-extract studied here, and they characterize single-sorbent systems optimized for capacity rather than for selective separation. The lower operating capacity of the present system is therefore an expected consequence of the dilute, multicomponent real feed, and is not a measure of the intrinsic affinity of the sorbent.

The advantage of the present system lies not in absolute capacity but in selectivity and in its operation of a genuine industrial solution. The spatially separated Cellulose-PEI-GA: Lewatit MonoPlus SP112H pair preferentially binds rhenium over molybdenum with a separation factor β(Re/Mo) of 9–15 across the studied compositions, comparable to the separation factors reported for dedicated Mo/W systems (β ≈ 108 for D301@TA, but for a different pair). In contrast to single-resin sorbents, the interpolymer arrangement exploits the cooperative interaction between the sulfonic groups of the cation exchanger and the protonated amino groups of the functionalized cellulose, which maximizes uptake at the balanced 3:3 ratio. The same cooperative principle was previously demonstrated for the PMAA: P4VP interpolymer system, where the 3:3 and 2:4 ratios gave rhenium extraction above 90% and molybdenum extraction reaching 94% from model solutions; the present work extends this approach to industrially functionalized cellulose and, importantly, to a real ammoniacal re-extract obtained after solvent-extraction pre-treatment. Overall, the proposed system is positioned as a selective, regenerable sorbent designed to operate within an integrated hydrometallurgical scheme, rather than as a high-capacity bulk sorbent. Its combination of process-level selectivity (solvent extraction) and sorbent-level selectivity (the interpolymer pair) is the principal practical advantage over the single-sorbent materials listed in Table 15.

Beyond the high sorption efficiency demonstrated in this study, the proposed interpolymer system represents a promising polymer-based material for environmentally oriented hydrometallurgical processes. Selective recovery of rhenium and molybdenum from acidic industrial solutions contributes to reducing the environmental impact of metallurgical wastes while improving the utilization of critical raw materials. The developed approach supports sustainable resource management through the recovery of valuable metals from secondary resources and may therefore be considered a promising strategy for environmentally friendly separation technologies.

## 4. Conclusions

In this work, an interpolymer system combining the anion-exchanging composite Cellulose-PEI-GA with the strong-acid cation exchanger Lewatit MonoPlus SP112H was developed and evaluated for the selective recovery of rhenium from a real industrial solution derived from copper-smelter lead dust. Because the feed contained rhenium at a low level against a large excess of competing cations and sulfate, a two-stage pre-treatment was applied: solvent extraction with a trialkylamine-2–ethylhexanol–kerosene system, which also co-precipitated part of the Cu, Zn, and As at the phase boundary as an interfacial crud, followed by ammonia stripping to give a re-extract containing 5.49 mg L^−1^ Re with a strongly reduced level of interfering metals.

The Cellulose-PEI-GA composite was prepared by grafting polyethyleneimine onto microcrystalline cellulose and crosslinking it with glutaraldehyde through Schiff base formation. The structure and the operating mechanism of the interpolymer system were confirmed by complementary methods. Nitrogen adsorption showed a progressive development of the mesoporous texture, with the specific surface area rising from 1.93 to 8.62 m^2^ g^−1^ after sorption. SEM-EDS located rhenium on the cellulose component (0.2 wt%) but not on the resin, where iron was retained instead, while FTIR revealed a perrhenate Re=O band at 894.9 cm^−1^ on the loaded cellulose and no such band on the resin. Together, these results demonstrate that the two phases act in a complementary way: the protonated amino groups of the modified cellulose bind the anionic perrhenate, whereas the cation exchanger retains competing cations and sustains the acidic environment required for protonation.

Sorption was governed by the composition of the interpolymer pair. The highest uptake of both metals was obtained at an equal Lewatit-to-cellulose ratio of 3:3, reflecting the mutual activation of the two phases. Under the optimal conditions (168 h), rhenium recovery reached 77.7% with a distribution coefficient of 3678 mL g^−1^ and an uptake of 4.51 mg g^−1^, whereas molybdenum was retained to a lesser extent under the same conditions (24.0%, Kd = 333 mL g^−1^, q = 18.03 mg g^−1^). The pseudo-first-order and pseudo-second-order models described the uptake kinetics equally well, while the Elovich model indicated a markedly higher initial sorption rate for rhenium than for molybdenum (α = 950.5 vs. 1.49 mg·g^−1^·h^−1^). Both the Weber–Morris and Boyd analyses resolved into two linear stages (24–72 h and 72–168 h) with non-zero intercepts, indicating that film diffusion dominates the initial stage while film and intraparticle diffusion operate together as the system approaches equilibrium. The system was markedly selective towards rhenium, with a Re/Mo separation factor of 8.8–15.5 across the compositions studied, a preference that arises from the lower charge density and weaker hydration of the singly charged perrhenate relative to the doubly charged molybdate.

Overall, the proposed scheme provides selectivity at two levels acting in series—the solvent-extraction stage removes the bulk of the competing cations, and the interpolymer sorption then concentrates rhenium with a high Re/Mo separation factor. The interpolymer system was also readily regenerable: elution with 0.5 M HCl recovered 91.4% of the sorbed rhenium and 89.1% of the sorbed molybdenum at the optimal 3:3 composition, confirming that the strong affinity responsible for the observed selectivity does not compromise reversibility and support the sorbent’s suitability for repeated sorption–desorption cycles. By design, PEI is covalently anchored to the cellulose matrix through Schiff base crosslinking with glutaraldehyde (Section 1), which is expected to limit polymer leaching during acidic, sulfate-rich sorption–desorption cycling; the present study, however, evaluated only a single sorption–desorption cycle, and quantitative assessment of PEI leaching, structural stability over multiple regeneration cycles, and sorbent cost under prolonged industrial operation remain to be established in future work. This combination offers a practical route to the selective recovery of rhenium from the complex acidic liquors of the Zhezkazgan copper smelter and, more broadly, from similar multicomponent metallurgical solutions.

The proposed polymer-based interpolymer system demonstrates considerable potential for the environmentally sustainable recovery of critical metals from acidic industrial solutions. The results provide a foundation for designing advanced polymer materials for environmental remediation and resource recovery, in line with the principles of sustainable hydrometallurgy and circular resource use.

## Figures and Tables

**Table 1:** Compositional analysis of the industrial sulfuric acid solution: major components.

Re (mg L^−1^)	Al (g L^−1^)	Mo (mg L^−1^)	Cu (g L^−1^)	Pb (g L^−1^)	Zn (g L^−1^)	Fe (g L^−1^)	SO_4_ (g L^−1^)	K (g L^−1^)	Bi (g L^−1^)	Cl (g L^−1^)	Cd (g L^−1^)	Si (g L^−1^)	As (g L^−1^)
22.73	0.389	143.26	29.709	0.016	50.7	2.31	128.5	2.736	0.713	0.176	5.44	1.402	0.16

Note: Re and Mo in Mg L^−1^; all Other Components in G L^−1^.

**Table 2:** Compositional analysis of the industrial sulfuric acid solution: minor components.

Mn (g L^−1^)	Co (g L^−1^)	Na (g L^−1^)	Ca (g L^−1^)	Ni (mg L^−1^)	Se (mg L^−1^)	P (g L^−1^)
0.027	0.003	0.247	0.58	0.064	0.0015	3.65

Note: Ni and Se in mg L^−1^; all others in g L^−1^. pH measured potentiometrically at 25 °C.

**Table 3:** XRF analysis of lead dust.

Element	Content, %	Element	Content, %
O	28.510	Fe	0.644
Al	0.049	Cu	4.177
Si	0.261	Zn	3.916
P	0.011	As	1.558
S	6.602	Cd	0.924
Cl	0.111	Pb	52.990
K	0.150	Bi	0.097

Note: Contents are given in wt.%. The sum is not normalized to 100% owing to unaccounted light elements (H, C, N) not detectable by XRF. Values are semi-quantitative.

**Table 4:** Chemical composition of lead dust, wt.%.

Re	Mo	K	Cu	Bi	Fe	Cd	Zn	Al	Ca	Ni	Se
0.0211	0.138	0.3637	9.8367	0.1897	5.2294	0.3192	3.8292	0.5154	0.3210	0.0348	0.0049
As		S		Cl^−^		Pb		SiO_2_		P	
2.85		14.350		0.1065		27.98		0.94		0.21	

Note: Contents are given in wt.% (bulk composition after complete dissolution). Silicon is reported as SiO_2_; chlorine as Cl^−^.

**Table 5:** XRF analysis of the crud (interfacial suspension) isolated during rhenium extraction.

Element	O	Na	Al	Si	P	S	Cl	K	Cu	Fe	Ca	Mn	Zn	As	Mo	Cd	Re
Content wt.%	30.890	1.894	0.159	7.602	0.024	13.949	0.020	0.484	11.334	1.187	0.267	0.027	15.160	8.021	0.297	0.322	0.119

**Table 6:** Rhenium and molybdenum concentrations in extraction and re-extraction fractions.

No.	Fraction	Solution Description	Volume, mL	[Re], mg L^−1^ (Chem.)	[Re], mg L^−1^ (Spectroph.)	[Mo], mg L^−1^ (Chem.)	[Mo], mg L^−1^ (Spectroph.)
1	Industrial feed solution *	Pale blue, transparent	1000	22.73	—	143.26	—
2	Raffinate (post-extraction)	Deep blue, transparent	975	5.084	5.102	23.320	23.456
3	Loaded organic phase	Light yellow–brown	65	10.155	10.204	111.24	112.04
4	Re-extract	Pale yellow, slight turbidity	975	5.482	5.494	71.16	71.23
5	Stripped organic	Light yellow, residual turbidity	65	0.583	0.588	7.8	7.91

Note: *—the industrial feed solution was not analyzed by spectrophotometry (chemical method only). The re-extract (5.494 mg L^−1^ Re) was directed to the sorption stage.

**Table 7:** Experimental conditions for sorption studies.

Parameter	Value/Condition
Sorption vessel	Erlenmeyer conical flasks, 250 mL
Agitation	Orbital shaker, 150 rpm
Sorbent dose	4.0 g L^−1^ total IPS loading (0.5 g each component per 250 mL solution; equivalent to 2 g L^−1^ per component
Contact time	0.5–24 h
Temperature	298–328 K (25–55 °C)
pH range	1.0–10.0 (adjusted with 1 M H_2_SO_4_ or 1 M NaOH)
Initial Re and Mo concentration	0.01–2.0 g L^−1^
Phase separation	Filtration through 0.45 μm membrane
Metal analysis	ICP-OES (PerkinElmer Optima 830)
Number of replicates	3 (RSD ≤ 2%)

**Table 8:** EDS acquisition conditions.

Parameter	Sample 1 (Pristine Cellulose)	Sample 2 (Modified Cellulose)	Sample 3 (After Sorption)
Measurement site	Site 5	Site 6	Site 9
Accelerating voltage, kV	5	10	20
Total counts	61,232	300,382	242,035
Mean count rate, cps	684	4130	891
Acquisition time, s	90	73	272

Note: The accelerating voltage was selected individually for each sample based on the matrix composition and anticipated elemental range.

**Table 9:** Surface elemental composition of the samples from EDS (at.%/wt.%).

Element	Line	Sample 1 Pristine Cellulose at.%/wt.%	Sample 2 Modified Cellulose at.%/wt.%	Sample 3 After Sorption at.%/wt.%
C	K	30.8/25.0	42.3/36.5	45.8/39.1
O	K	69.2/75.0	36.9/42.6	41.4/46.9
N	K	-	20.8/20.9	12.4/12.4
S	K	-	-	0.1/0.2
Fe	K	-	-	0.3/1.0
Re	L	-	-	0.0/0.2
Mo	L	-	-	0.0/0.2

Note: “-” element not detected in this sample; uncertainties correspond to one standard deviation.

**Table 10:** Surface elemental composition of the Lewatit MonoPlus SP112H resin before and after sorption (at.%/wt.%).

Element	Line	Before Sorption (At. %/wt.%)	After Sorption (At. %/wt.%)
C	K	59.9/49.4	56.5/43.7
O	K	34.1/37.4	33.5/34.6
S	K	6.0/13.2	8.8/18.3
Na	K	-	0.1/0.2
Al	K	-	0.1/0.2
Si	K	-	0.4/0.8
Fe	K	-	0.6/2.2

Note: ‘-’ element not detected.

**Table 11:** Recovery (R), uptake (q) and distribution coefficients (K_d_) of Re(VII) and Mo(VI) at 168 h, and the Re/Mo separation factor β for the studied interpolymer compositions (referred to the total IPS mass; the 3:3 composition in bold).

Ratio	R(Re) %	q(Re) mg g^−1^	Kd(Re) mL g^−1^	R(Mo) %	q(Mo) mg g^−1^	Kd(Mo) mL g^−1^	β(Re/Mo)
6:0	11.6	0.67	139	1.5	1.13	16	8.84
5:1	54.4	3.16	1262	8.3	6.25	96	13.20
4:2	61.2	3.56	1668	10.4	7.83	123	13.59
**3:3**	**77.7**	**4.51**	**3678**	**24.0**	**18.03**	**333**	**11.04**
2:4	68.5	3.98	2300	16.7	12.58	212	10.86
1:5	64.3	3.74	1905	10.4	7.83	123	15.54
0:6	47.5	2.76	957	7.3	5.50	83	11.53

**Table 12:** Kinetic parameters for Re(VII) sorption (non-linear PFO/PSO; q in mg g^−1^; 3:3 in bold).

Ratio	q_e_,_exp_	PFO q_e_	PFO k_1_	PFO R^2^	PSO q_e_	PSO k_2_	PSO R^2^
6:0	0.676	0.625	0.0261	0.738	0.778	3.34 × 10^−2^	0.691
5:1	3.163	3.115	0.0532	0.923	3.390	2.75 × 10^−2^	0.914
4:2	3.555	3.504	0.0748	0.967	3.680	4.95 × 10^−2^	0.851
**3:3**	**4.512**	**4.390**	**0.0764**	**0.874**	**4.622**	**3.92 × 10^−2^**	**0.898**
2:4	3.977	3.910	0.1056	0.919	4.014	9.73 × 10^−2^	0.913
1:5	3.734	3.565	0.0742	0.534	3.783	4.26 × 10^−2^	0.686
0:6	2.761	2.707	0.0348	0.973	3.132	1.42 × 10^−2^	0.927

**Table 13:** Kinetic parameters for Mo(VI) sorption (non-linear PFO/PSO; q in mg g^−1^; 3:3 in bold).

Ratio	q_e_,_exp_	PFO q_e_	PFO k_1_	PFO R^2^	PSO q_e_	PSO k_2_	PSO R^2^
6:0	1.178	1.201	0.0103	0.681	1.842	3.83 × 10^−3^	0.680
5:1	6.321	6.745	0.0151	0.829	9.604	1.19 × 10^−3^	0.811
4:2	7.902	8.315	0.0196	0.840	11.114	1.49 × 10^−3^	0.804
**3:3**	**18.030**	**18.288**	**0.0285**	**0.951**	**22.012**	**1.44 × 10^−3^**	**0.899**
2:4	12.611	12.529	0.0294	0.943	14.944	2.26 × 10^−3^	0.899
1:5	7.884	7.887	0.0298	0.941	9.389	3.66 × 10^−3^	0.890
0:6	5.556	5.401	0.0380	0.948	6.155	8.39 × 10^−3^	0.892

**Table 14:** Desorption efficiencies of rhenium and molybdenum.

Lewatit MonoPlus SP112H: Cellulose-PEI-GA	Re (%)	Mo (%)
6:0	67.1	66.4
5:1	75.2	73.1
4:2	83.3	82.6
**3:3**	**91.4**	**89.1**
2:4	85.7	84.1
1:5	78.5	77.2
0:6	70.6	69.9

**Table 15:** Comparison of the rhenium- and molybdenum-sorption performance of the Cellulose-PEI-GA: Lewatit MonoPlus SP112H interpolymer system with anion-exchange resins and interpolymer sorbents reported in the literature *.

Sorbent	Functional Group	Target/Medium	Capacity (mg g^−1^)	R, %	Selectivity/Notes	Ref.
Purolite A530E/A532E	Bifunctional quaternary amine	${ReO}_{4}^{-}$/Aqueous, pH 1–13	707/446	-	High selectivity over ${NO}_{3}^{-}$, ${SO}_{4}^{2-}$	[63]
Anion-exchange resin D318	Quaternary amine	Re(VII)/aqueous	437	-	Langmuir monolayer	[64]
Novel anion-exchange resins	Amine	Re(VII)/acidic (Cu-Mo by-product sim.)	up to 435	-	Higher than commercial resins	[65]
4-amino-1,2,4-triazole resin	Triazole/amine	Re(VII)/HAc–NaAc, pH 2.6	354	-	-	[66]
Purolite A170	Complex amine	Re/sulfate	~100	-	Weak-based, hydrophobic anions	[5]
AM-p-2 (macroporous SDV)	Dimethylammonium	Re/sulfate (dynamic)	81.0	>90	Recovery >90% after ~1500 BV	[67]
A920 strong-base resin	Quaternary amine	Re/U co-sorption	16.5	-	Ion-exchange mechanism	[68]
D301 (weak base)	Tertiary amine	Mo(VI)/acidic leach	up to 464	-	Particle-diffusion control	[69]
IRA-400	Quaternary amine	Mo(VI)/pH 6	208	-	Mo7O24/MoO42−	[70]
Modified D301 (D301@TA)	Assembled tertiary amine	Mo/tungstate, pH 7.2	414–428	-	β(Mo/W) ≈ 108	[71]
PMAA:P4VP interpolymer (3:3/2:4)	Carboxyl + pyridine	Re, Mo/Model solution	-	R > 90% (Re), up to 94% (Mo)	Cooperative IPS effect	[72]
**This work: Cellulose-PEI-GA: Lewatit MonoPlus SP112H (3:3)**	**Protonated amino (PEI) + sulfonic**	**Re, Mo/real ammoniacal re-extract**	4.51 (Re); 18.03 (Mo) *	**77.7% (Re); 24% (Mo)**	**β(Re/Mo) = 9–15; real industrial feed**	-

Note: * Operating capacities from a real ammoniacal re-extract (C_0_ ≈ 25 mg L^−1^ Re), referred to the total IPS mass; not directly comparable with Langmuir saturation maxima obtained from concentrated model solutions.

**Figure A2:**
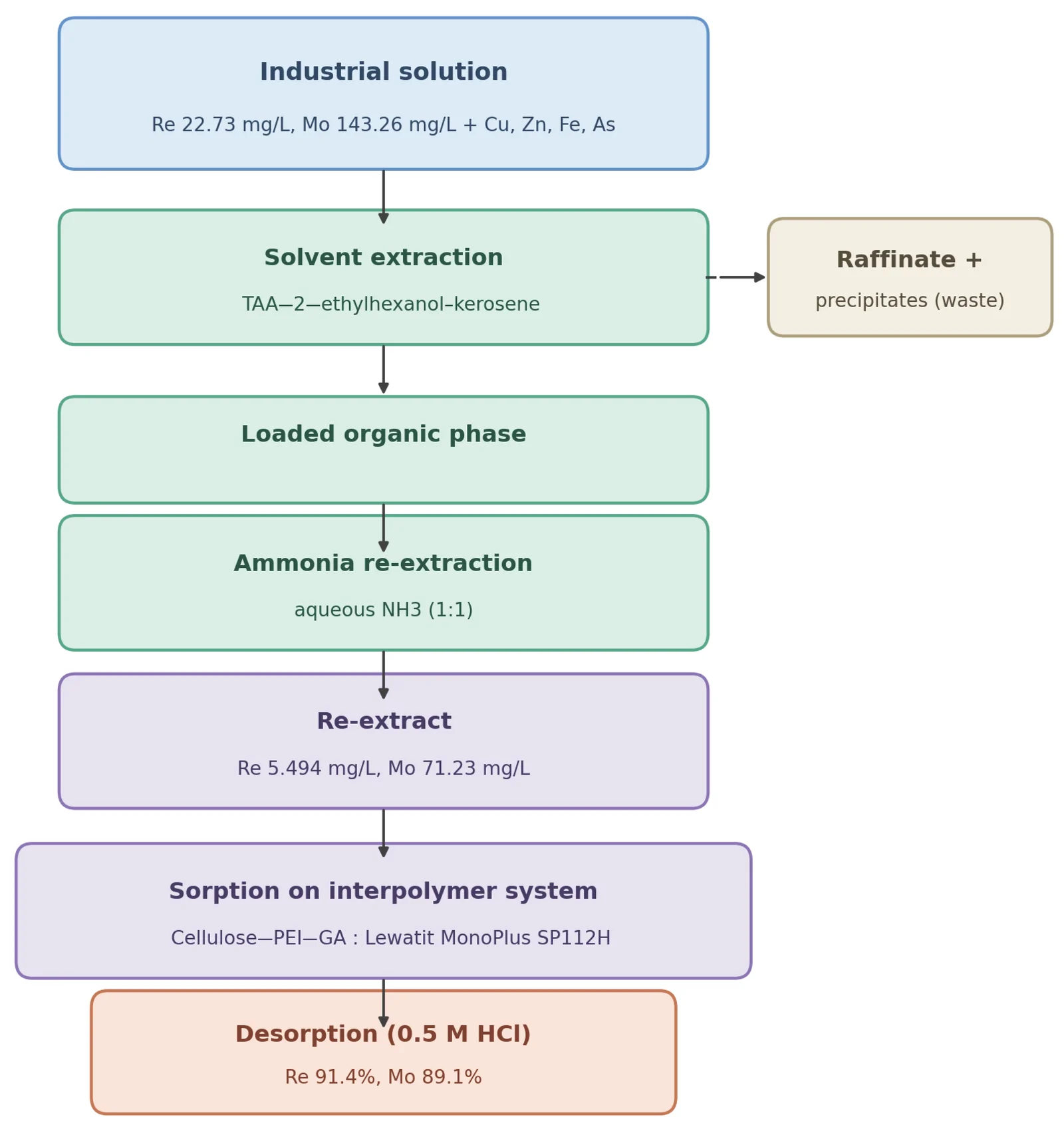
Schematic flow diagram of the laboratory rhenium recovery process from the industrial solution: solvent extraction (TAA-2–ethylhexanol–kerosene), ammoniacal re-extraction, sorption on the Cellulose–PEI-GA: Lewatit MonoPlus SP112H interpolymer system, and desorption with HCl.

**Figure A3:**
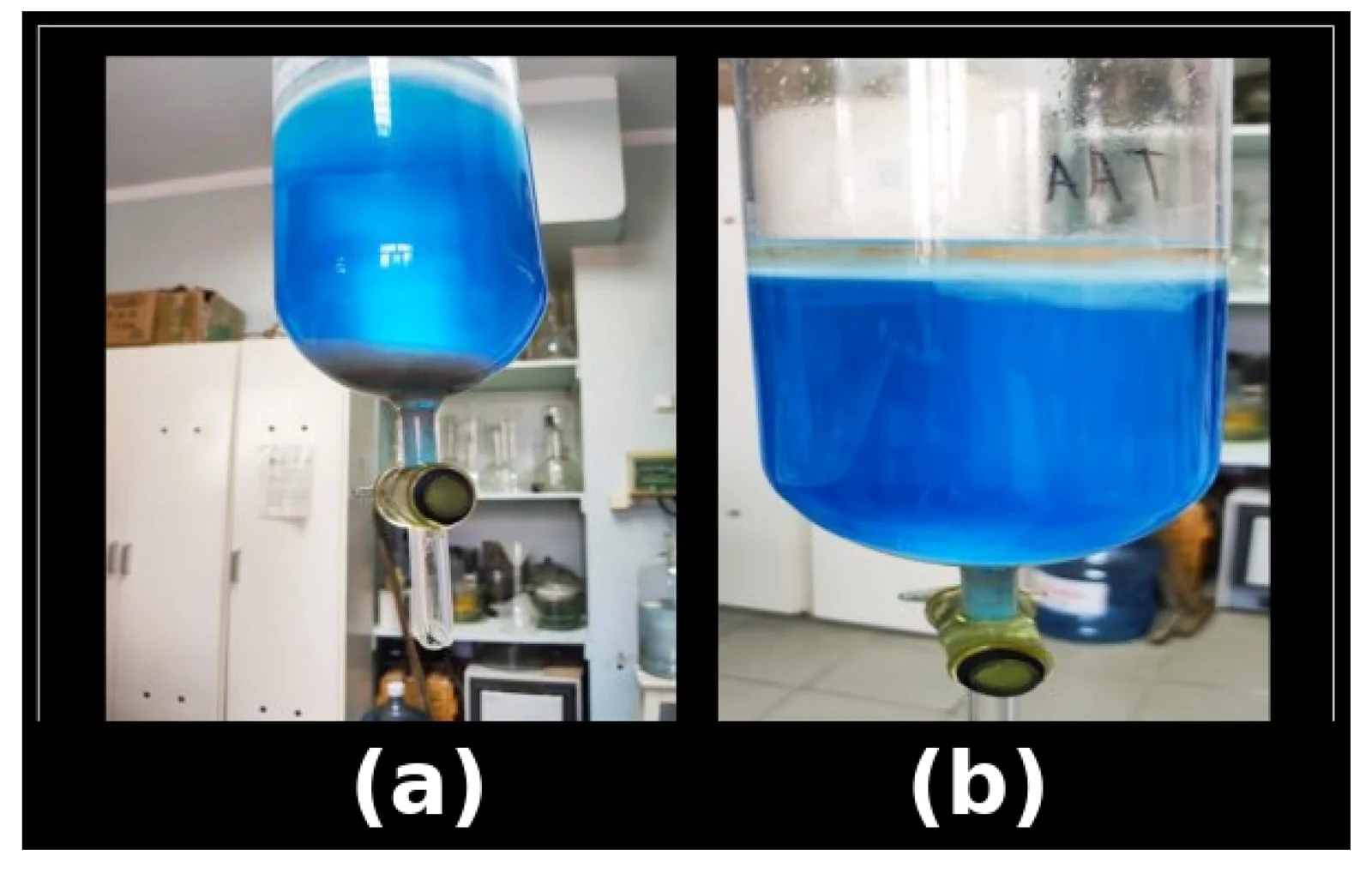
Liquid–liquid extraction of rhenium in a separating funnel after phase separation: (**a**) the upper loaded organic phase with the white interfacial precipitate (“crud”) at the phase boundary above the clear blue raffinate; (**b**) the same system after removal of the interfacial crud, showing the separated organic and aqueous phases without visible precipitate.

## Data Availability

All data supporting the findings of this study are included within the article.

## Supplementary Materials

The following supporting information can be downloaded at: https://www.mdpi.com/article/10.3390/polym18161943/s1. Section S1: Effect of pH on rhenium and molybdenum sorption by the Cellulose-PEI-GA: Lewatit MonoPlus SP112H interpolymer system; Table S1: Residual concentrations and sorption efficiencies of Re and Mo on the Cellulose-PEI-GA: Lewatit MonoPlus SP112H IPS as a function of solution pH; Figure S1: Effect of solution pH on the sorption efficiency of Re(VII) and Mo(VI) by the Cellulose-PEI-GA/Lewatit MonoPlus SP112H interpolymer system (industrial solution, Shymkent; C_0_(Re) = 21.806 mg L^−1^, C_0_(Mo) = 79.81 mg L^−1^; IPS 1.0 g/100 mL). Error bars represent the standard deviation (SD below 2%); Table S2: Rhenium and molybdenum speciation and IPS functional group state as a function of pH; Section S2. BET Analysis of the Porous Structure of the Cellulose-PEI-GA Sorbent; Table S3. Textural characteristics of the Cellulose-PEI-GA samples studied from BET analysis; Section S3. BET Analysis of the Porous Structure of the Lewatit MonoPlus SP112H Resin; Table S4. Textural characteristics of the Lewatit MonoPlus SP112H resin from BET analysis.
