# Supplementary material for: Selective Sorption of Rhenium and Molybdenum Oxoanions Using an Interpolymer System Based on Functionalized Cellulose and Lewatit Monoplus SP112H in Acidic Aqueous Media
Source: Polymers (Basel). 2026 Aug 8;18(16):1943. doi: 10.3390/polym18161943 (PMC13516062; doi:10.3390/polym18161943)
Supplement: Supplementary file 1 [file polymers-18-01943-s001.zip › polymers-4458454-supplementary.pdf]

Supplementary Materials

Section S1. Effect of pH on Rhenium and Molybdenum Sorption by the Cellulose-PEI-GA : Lewatit MonoPlus SP112H Interpolymer System

Solution pH is among the most influential variables governing ion-exchange sorption, as it simultaneously controls the protonation state of the sorbent functional groups, the speciation of metal ions in solution, and competitive effects from H^+ and OH^-. The present section examines how pH affects the sorption of rhenium and molybdenum from an industrial sulfuric acid leach liquor (blue solution, Shymkent) onto the interpolymer system (IPS) comprising Cellulose-PEI and Lewatit MonoPlus SP112H. The industrial feed solution had 22.73 mg L^{-1} Re and 143.26 mg L^{-1} Mo at pH 1.25. The pH of the downstream processing reference ammoniacal re-extract was 11.98. An equal mass of both IPS components (Cellulose-PEI-GA and Lewatit MonoPlus SP112H, 0.5 g each) was contacted with 100 mL of the industrial solution, adjusted to the target pH. The pH was adjusted from 2 to 10 with 1 M NaOH or 1 M HCl. The sorption efficiency R (%) was calculated using the following formula: $R = (C_0 - C_e)/C_0 \times 100\%$ (1), where C_0 is the initial concentration (mg L^{-1}), and C_e is the residual concentration at equilibrium (mg L^{-1}). The specific sorption capacity was calculated by $q = (C_0 - C_e)\ V / m$ (mg g^{-1}), where V = 0.1 L, m = 1.0 g (total IPS mass). Table S1 presents a mechanistic explanation for the observed trends by summarizing the dominant Re and Mo species and the protonation state of the IPS functional groups as a function of pH.

Table S1. Residual concentrations and sorption efficiencies of Re and Mo on the Cellulose-PEI-GA : Lewatit MonoPlus SP112H IPS as a function of solution pH

pH	Ce(Re), mg L^{-1}	Ce(Mo), mg L^{-1}	R(Re), %	R(Mo), %	q(Re), mg g^{-1}	q(Mo), mg g^{-1}
Feed solution	22.73	143.26	–	–	–	–
2	21.656	1.928	4.73	98.65	0.107	14.133
4	20.710	8.771	8.89	93.88	0.202	13.449
6	10.027	15.980	55.89	88.85	1.270	12.728
8	5.013	28.697	77.95	79.97	1.772	11.456
10	4.570	32.705	79.89	77.17	1.816	11.056

Note: C_0(Re) = 22.73 mg L^{-1}; C_0(Mo) = 143.26 mg L^{-1}; V = 100 mL; m(IPS) = 1.0 g. q - specific sorption capacity (mg g^{-1}) = $(C_0 - C_e)V/m$. Bold entries indicate the best sorption performance for each ion.

Overall trends. The data in Table S1 reveal opposing pH dependencies for Re and Mo sorption (Fig. S1).

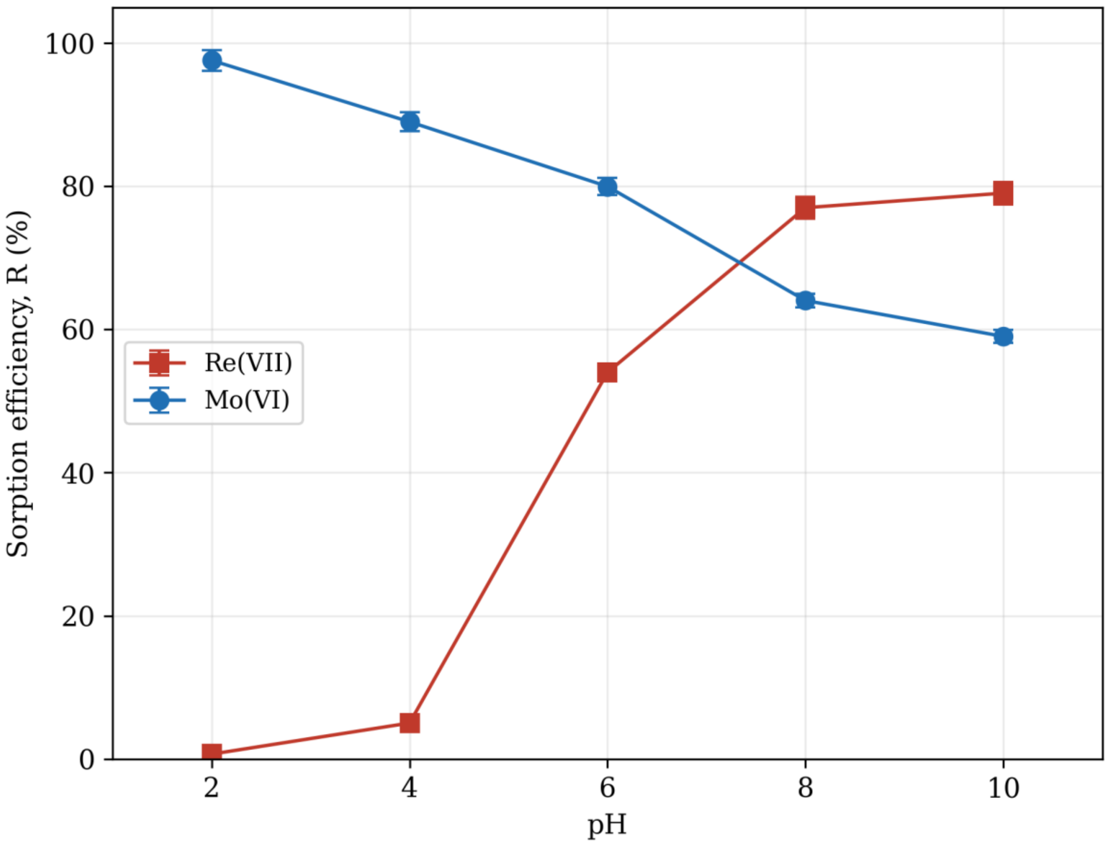

Figure S1. Effect of solution pH on the sorption efficiency of Re(VII) and Mo(VI) by the Cellulose-PEI-GA / Lewatit MonoPlus SP112H interpolymer system (industrial solution, Shymkent; C_0(Re) = 21.806 mg L^{-1}, C_0(Mo) = 79.81 mg L^{-1}; IPS 1.0 g / 100 mL). Error bars represent the standard deviation (SD below 2%).

Molybdenum extraction decreases monotonically with rising pH, from a maximum of 98.65% at pH 2 to 77.17% at pH 10. Rhenium shows the opposite behavior, increasing from only 4.73% at pH 2 to 79.89% at pH 10. This difference is due to the very different solution chemistry of the two metals and their different ways of interacting with the IPS functional groups.

Molybdenum:

Molybdenum removal at pH 2 is 98.65%, q = 14.133 mg/g, which is almost quantitative, with selective uptake at acidic pH. Under strongly acidic conditions, molybdenum is predominantly present as highly charged polymolybdate anions ($[Mo_7O_{24}]^{6-}$, $[Mo_8O_{26}]^{4-}$, etc.), which carry a large negative charge and show high affinity for the fully protonated amine groups of PEI (-NH_3^+, -NH_2^+). At pH 4, the Mo removal is still high (93.88% removal, q = 13.449 mg/g), but the progressive transformation of polymolybdate into less charged species such as $HMoO_4^-$ and MoO_4^{2-} reduces the driving force for uptake. Further increase to pH 6–10 shifts molybdenum predominantly to MoO_4^{2-} form. Competition of hydroxyl ions increases, and Mo removal decreases to 88.85% (pH 6), 79.97% (pH 8), and 77.17% (pH 10).

Rhenium: selective uptake under alkaline conditions.

Unlike molybdenum, the perrhenate ion, ReO_4^-, is stable across the entire pH range studied and does not undergo speciation changes. The very low Re removal at pH 2 (4.73%) is due to two simultaneous effects: first, at strongly acidic pH, all available PEI binding sites are occupied by highly charged polymolybdate anions, leaving virtually no capacity for ReO_4^-; second, the high sulfate and/or chloride concentration introduced with H_2SO_4/HCl used for acidification competes effectively for the same anion-exchange sites. As pH increases, the amine groups are less protonated, polymolybdate is converted to less charged species, competitive displacement is reduced, and ReO_4^- is extracted in significant amounts. The Re removal is 55.89 % (q = 1.270 mg/g) at pH 6, 77.95 % (q = 1.772 mg/g) at pH 8, and 79.89 % (q = 1.816 mg/g) at pH 10.

Selectivity and competition effects.

The results indicate a fundamental conflict between the optimal pH windows for Re and Mo recovery. Maximum Mo extraction occurs at pH 2, whereas maximum Re extraction occurs at pH 8-10. At pH 6, both ions are extracted together, 55.89 % Re and 88.85 % Mo, but neither is at its individual optimum. This suggests that a practical pH selective fractionation could be possible: a two-step procedure, where sorption at pH 2 leads to a selective trapping of Mo followed by sorption at pH 8-10 to concentrate Re, or a stepwise desorption at different pH values, could allow the separate recovery of the two metals.

Table S2. Rhenium and molybdenum speciation and IPS functional group state as a function of pH

pH	Re species	Mo species	PEI charge state	Dominant mechanism
< 2	ReO_4^-	$Mo_7O_{24}^{6-}$ (polymo); H_2MoO_4	$-NH_3^+$ (strongly protonated)	Weak Re exchange; Mo polymerization/exchange
2–4	ReO_4^-	$HMoO_4^-$; $Mo_7O_{24}^{6-}$	$-NH_2^+$ / $-NH_3^+$	Active anion exchange of high-charge polymolybdate; Re weak
4–7	ReO_4^-	MoO_4^{2-} / $HMoO_4^-$	$-NH_2^+-$; partly $-NH_2$	Mixed: Mo exchange decline; Re exchange increasing
7–10	ReO_4^-	MoO_4^{2-}	$-NH_2$ (neutral)	Re coordination via $-NH_2$; Mo exchange suppressed by OH^- competition
> 10	ReO_4^-	MoO_4^{2-}	$-NH_2$ (deprotonated)	Overall exchange reduced; ammonia re-extract (pH 11.98) desorbs Mo

Note: Mo speciation from Pourbaix diagrams for the Mo-H_2O system; Re speciation from Greenwood & Earnshaw, Chemistry of the Elements; PEI protonation based on pK_a values: primary amines ~8-10, secondary ~6-8, tertiary ~3–5

Role of Lewatit MonoPlus SP112H and Cellulose-PEI-GA within the IPS (corrected).

Lewatit MonoPlus SP112H is a strong acid cation exchange resin based on a styrene-divinylbenzene matrix bearing sulfonic acid ($-SO_3H$) groups; it does not contain amine functionality and does not itself perform anion exchange. Because $-SO_3H$ is a strong acid group, it remains essentially fully dissociated across the entire pH range studied (2-10), so its own ion-exchange behavior changes little with pH. Within the IPS, SP112H's role is therefore complementary rather than co-active in anion binding: it exchanges background cations (H^+, Na^+ and other cations present after pH adjustment), helping to buffer local pH and charge balance at the sorbent surface and contributing structural/textural support to the interpolymer network. All of the pH-dependent anion-exchange chemistry described above for the polymolybdate/molybdate species ($Mo_7O_{24}^{6-}$, $HMoO_4^-, MoO_4^{2-}$) and for perrhenate (ReO4-) is carried out by the primary, secondary, and tertiary amine groups of Cellulose-PEI-GA alone. The protonation state of these amines - from fully protonated $-NH_3^+$/$-NH_2^+$ at low pH (favoring high-charge polymolybdate uptake) to largely neutral -NH2 at high pH (favoring ReO_4^- coordination with reduced competition) - is what generates the pH-dependent selectivity switch between Mo and Re summarized in Table S2. The IPS's effective operating window therefore reflects the pH-dependent amine chemistry of the PEI-GA component, with SP112H providing a supporting cation-exchange/charge-balancing function rather than a second, independently pH-switching anion-exchange site.

Relevance to the industrial process.

The native industrial feed solution has pH 1.25, placing it close to the regime of maximum Mo sorption (98.65% at pH 2) and minimal Re uptake (4.73% at pH 2). The ammoniacal re-extract, at pH 11.98, falls in the range where rhenium extraction approaches 80% (79.89% at pH 10) and Mo uptake is comparatively lower (~77%). These observations have direct process implications: (a) direct sorption from the untreated acidic feed (pH ~1.25) is most suitable for Mo recovery; (b) for preferential or combined Re recovery, neutralization of the solution to pH 8-10 prior to sorption, or a two-stage sorption scheme is advisable.

Conclusions.

Solution pH exerts a decisive influence on the sorption selectivity of the Cellulose-PEI-GA : Lewatit MonoPlus SP112H interpolymer system towards Re and Mo. At pH 2-4, molybdenum is preferentially extracted by forming highly charged polymolybdate anions and interacting with protonated PEI amine groups (Rmax = 98.65% at pH 2; q = 14.133 mg/g). Rhenium is extracted most efficiently at pH 8-10 as competitive displacement by polymolybdate diminishes and PEI anion-exchange sites remain accessible (Rmax = 79.89% at pH 10; q = 1.816 mg/g). A compromise pH of 6 has been identified at which both ions are simultaneously recovered with acceptable efficiency (Re: 55.89%; Mo: 88.85%). These findings provide a mechanistic basis for the design of a pH-controlled sequential sorption scheme for the separate concentration of rhenium and molybdenum.

Section S2. BET Analysis of the Porous Structure of the Cellulose-PEI-GA Sorbent

Table S3. Textural characteristics of the Cellulose-PEI-GA samples studied from BET analysis

Parameter	Sample No. 1 (Pristine cellulose)	Sample No. 2 (Cellulose-PEI-GA)	Sample No. 3 (No. 2 after Re sorption)
Specific surface area (BET, multipoint), m^2/g	1.9339	2.4169	8.6228
Specific surface area (Langmuir), m^2/g	1.7866	2.1786	6.6278
External surface area (t-plot), m^2/g	1.9339	2.4169	8.6228
Internal (micropore) surface area (t-plot), m^2/g	0.0000	0.0000	0.0000
Mesopore surface area (αs-method), m^2/g	2.9970	3.5003	14.6200
Cumulative BJH surface area, ads. (d > 2 nm), m^2/g	2.2892	2.9426	11.9131
Total pore volume, cm3/g	0.010208	0.007889	0.011745
BJH pore volume (adsorption, d > 2 nm), cm^3/g	0.003984	0.008209	0.014937
BJH pore volume (desorption, d > 2 nm), cm^3/g	0.008806	0.008269	0.014713
Micropore volume (t-plot), cm^3/g	0.0000	0.0000	0.0000
Average pore radius (BET), nm	10.56	6.53	2.72
Average pore radius (BJH, ads.), nm	3.48	5.58	2.51
Most probable pore size (BJH, ads.), nm	2.33	2.33	2.34
Most probable pore size (BJH, des.), nm	2.33	2.34	2.38

Note: Sample No. 1 - pristine cellulose; No. 2 - cellulose modified with polyethyleneimine and cross-linked with glutaraldehyde (Cellulose–PEI–GA); No. 3 - Sample No. 2 after rhenium sorption from the re-extract.

Section S3. BET Analysis of the Porous Structure of the Lewatit MonoPlus SP112H Resin

Table S4. Textural characteristics of the Lewatit MonoPlus SP112H resin from BET analysis

Parameter	Sample No. 4 (before sorption)	Sample No. 5 (after sorption)
Specific surface area (BET, multipoint), m^2/g	7.2143	8.4643
Specific surface area (Langmuir), m^2/g	7.3113	8.2301
External surface area (t-plot), m^2/g	7.2143	8.4643
Internal (micropore) surface area (t-plot), m^2/g	0.0000	0.0000
Mesopore surface area (αs-method), m^2/g	6.3684	11.6439
Cumulative BJH surface area, ads. ($d > 2$ nm), m^2/g	5.8666	9.7796
Total pore volume, cm^3/g	0.065755	0.062601
BJH pore volume (adsorption, $d > 2$ nm), cm^3/g	0.059262	0.062474
BJH pore volume (desorption, $d > 2$ nm), cm^3/g	0.067162	0.063122
Micropore volume (t-plot), cm^3/g	0.0000	0.0000
Average pore radius (BET), nm	18.23	14.79
Average pore radius (BJH, ads.), nm	20.20	12.78
Most probable pore size (BJH, ads.), nm	46.91	3.16
Most probable pore size (BJH, des.), nm	27.08	29.82
